# Synergistic Enhancement of Electrical‐Thermal Properties in KH550/G‐POSS Functionalized BN/Epoxy Composites for High‐Frequency Transformer Insulation

**DOI:** 10.1002/advs.75952

**Published:** 2026-06-02

**Authors:** Jun Jiang, Shaojie Luo, Dezhi Cui, Zeya Huang, Shuai Jiang, Renli Fu, Yuan Wang

**Affiliations:** ^1^ Department of Electrical Engineering College of Automation Engineering Nanjing University of Aeronautics and Astronautics Nanjing China; ^2^ Liaocheng Power Supply Company State Grid Shandong Electric Power Company Shandong China; ^3^ College of Materials Science and Technology Nanjing University of Aeronautics and Astronautics Nanjing China; ^4^ Research Center for Membrane and Film Technology Department of Chemical Science and Engineering Kobe University Kobe Japan; ^5^ School of Mechanics and Engineering Science Peking University Beijing China

**Keywords:** BN@G‐POSS functionalization, electro‐thermal reliability, epoxy insulation composites, high‐frequency transformer, power electronic transformers

## Abstract

With the growing integration of renewable energy and DC power electronics, power electronic transformers (PETs) are vital in AC/DC hybrid grids. Their core component, high‐frequency transformers (HFTs), operates under severe electrical and thermal stresses, such as high dv/dt, high switching frequencies, and elevated temperatures that exceed the limits of conventional epoxy (EP) insulation. These limitations necessitate advanced materials with improved thermal conductivity and electro‐thermal stability for multi‐field conditions. To address this challenge, a synergistic enhancement strategy was proposed by integrating BN nanofillers functionalized through a two‐step KH550/G‐POSS modification. This interface design enables simultaneous regulation of dielectric behavior, heat transport pathways, and processing characteristics. An optimized formula with 7 wt.% G‐POSS content, and 20 vol% BN@G‐POSS nanofiller loading promises an improved thermal conductivity (492.5%) and a reduced viscosity (3493 mPa·s) compared to traditional BN/EP. Furthermore, the composite exhibits robust resistance against electro‐thermal degradation under a 100 kHz electric field, as demonstrated by a significantly elevated partial discharge inception voltage (increased by 266.5 V) and a remarkably attenuated total discharge amplitude (decreased by 256.5742 V). Overall, the BN@G‐POSS/EP system shows strong potential for next‐generation HFTs and PETs, underscoring the importance of interdisciplinary material design in addressing reliability challenges in modern power electronics.

## Graphical abstract

A BN@G‐POSS/EP nanocomposite is developed for high‐frequency transformer insulation in power electronic transformers. Through synergistic interface engineering, it simultaneously enhances thermal conductivity, reduces viscosity, and improves electro‐thermal stability. The material exhibits superior resistance to partial discharge under high‐frequency stress, offering a promising pathway toward reliable insulation in next‐generation AC/DC hybrid grids.

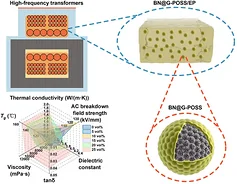

## 1 Introduction

The rapid growth of new energy generation, large‐scale direct current (DC) loads, and distributed power flow is accelerating the transition from conventional alternating current (AC) grids to next‐generation hybrid AC/DC power systems [1, 2, 3]. In this context, traditional industrial‐frequency transformers are increasingly inadequate in meeting the demands for flexible and efficient energy routing. Power electronic transformers (PETs), also known as solid‐state transformers (SSTs) [2, 4], have therefore emerged as a key class of intelligent power‐electronics equipment. By tightly integrating high‐frequency transformers (HFTs) with advanced power electronic conversion, PETs enable compact construction, high operational efficiency, and flexible control, making them indispensable in modern industrial applications including high‐speed electric traction, medium‐voltage hybrid power distribution, renewable energy grid‐connection, data centers powered by artificial intelligence computing, ultra‐fast electric vehicle charging infrastructure, and energy internet architectures [2, 5, 6, 7, 8].

As the core of PET systems, HFT is responsible for high‐frequency electrical isolation and energy transfer. Epoxy resin (EP, Figure S1) is the most widely adopted electrical encapsulation polymer in HFTs due to its high dielectric strength, room‐temperature processability, mechanical stability, and low cost [9, 10, 11]. However, the high‐frequency square‐wave voltage and the elevated thermal load confined within the compact transformer structure impose severe coupled electrical‐thermal stresses on EP [12, 13]. These stresses accelerate molecular degradation, while its intrinsically low thermal conductivity and rising high‐frequency dielectric loss can trigger insulation failure or even thermal runaway [14]. Therefore, enhancing heat dissipation while maintaining strong high‐frequency insulation is essential for the long‐term reliability of HFTs.

Introducing thermally conductive nanofillers is a practical and scalable approach for overcoming EP's low thermal conductivity. Among various fabrication approaches [15, 16], such as 3D structuring, interfacial modification engineering and segregated network formation, direct blending of thermally conductive nanofillers offers a robust and scalable route suitable for industrial production. For nanofillers, intrinsic thermal conductivity of the nanofillers is of crucial importance to determine the effective heat‐transport capability of the EP matrix. Accordingly, carbon‐based nanofillers such as graphene [17], carbon nanotubes [18], and diamond possess extremely high thermal conductivity [19], but their high electrical conductivity or high material cost severely limits their HFT application. In contrast, boron nitride (BN) combines high intrinsic thermal conductivity (about 400 W m^−1^ K^−1^) [20, 21, 22], excellent dielectric strength, and stable high‐frequency properties, makes it a highly suitable nanofiller for EP‐based insulation systems. Especially for hexagonal boron nitride (h‐BN) [23, 24, 25], its high aspect ratio and surface area is particularly attractive for constructing thermal conduction pathways in polymer matrices. Nevertheless, the severe interfacial incompatibility between inorganic BN and organic EP leads to phonon scattering, interfacial thermal resistance, and defect formation, which further aggravate dielectric loss and partial discharge (PD) under high electric fields [26, 27, 28]. While for the organic nanofiller cooperation, Wang et al. demonstrated that 1 wt.% POSS increased the DC breakdown strength of epoxy by 11.8% at 30°C and suppressed space charge accumulation, while 10 wt.% POSS significantly elevated the glass transition temperature (Tg) from 180°C to 222°C. Similarly, Shi et al. found that 5 wt.% Glycidyl POSS substantially enhanced volume resistivity and reduced low‐frequency dielectric loss during thermal aging. Furthermore, Aslam et al. showed that integrating 2.5 wt.% AP‐POSS yielded a 4.9% increase in DC breakdown strength and restricted charge carrier mobility by notably enhancing the interfacial crosslinking density (from 1.78 × 10^3^ mol/m^3^ to 2.31 × 10^3^ mol/m^3^). Given that the insufficient thermal conductivity of EP and the insulation degradation induced by electro‐thermal coupled stress are critical factors restricting the heat dissipation efficiency of HFTs and threatening their insulation reliability, this work focuses on the synergistic optimization of the electrical and thermal properties of EP tailored to the operating conditions and requirements of HFTs.

**FIGURE 1:**
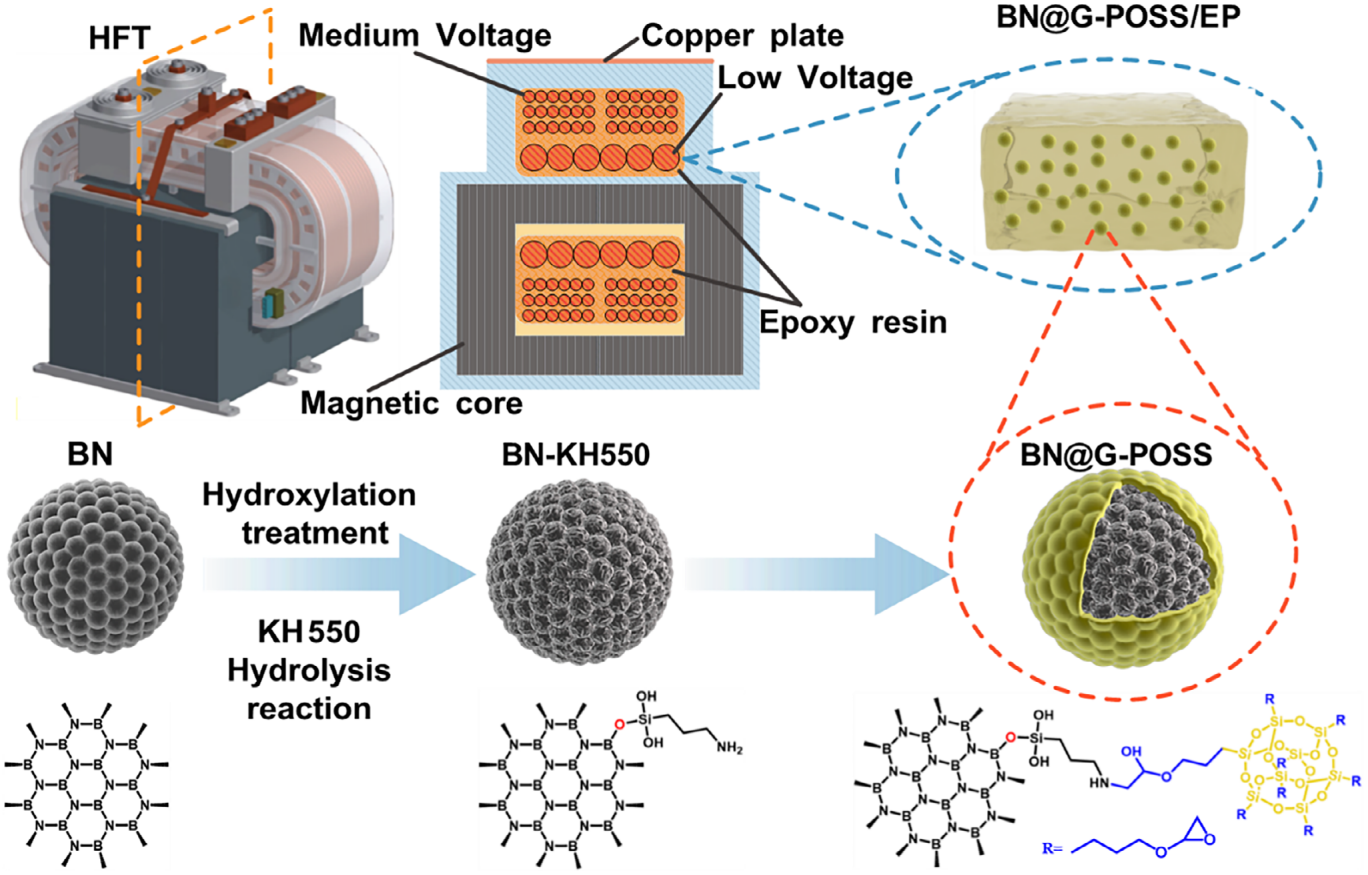
Fabrication of BN@G‐POSS/EP composite and its application in HFT encapsulation.

In this work, a two‐step surface modification strategy was reported. through sequential grafting reactions, γ‐aminopropyltriethoxysilane (KH550) was first introduced onto the BN surface and served as a molecular bridge. Subsequently, glycidyl‐functionalized polyhedral oligomeric silsesquioxane (G‐POSS) was grafted onto the KH550‐modified BN, forming a core‐shell structure with enhanced organic‐compatible characteristics [29, 30]. Each G‐POSS molecule contains eight epoxy groups [31], and their reaction with the amino groups on KH550‐modified BN produces abundant hydroxyl groups [32], markedly increasing the surface reactivity and improving the organic compatibility of BN (Figure 1). These newly generated functional groups can further participate in EP curing and form hydrogen bonds, thereby strengthening the interface and helping reduce dielectric loss at high frequencies. In addition, the cage‐like G‐POSS structure exhibits nanoscale effects and features multiple Si─O─Si bonds similar to SiO_2_, imparting excellent dielectric and insulation properties to the modified nanofillers [33, 34]. The resulting BN@G‐POSS/EP composites not only maintain outstanding electrical insulation but also exhibit markedly improved thermal conductivity. Comprehensive evaluation of thermal transport, dielectric response, and insulation characteristics confirms that the proposed composite effectively resolves the long‐standing trade‐off between thermal dissipation and high‐frequency insulation. This work provides a high‐insulation, high‐thermal‐conductivity epoxy encapsulation material with strong potential for long‐term industrial deployment in high‐frequency transformers and next‐generation power electronic systems.

## 2 Results and Discussion

### 2.1 Structural Characterization

Addressing the critical deficiencies in thermal conductivity and the accelerated insulation degradation of EP used in HFT potting under severe electric‐thermal stress, the synergistic modulation of electro‐thermal properties via high‐thermal conductivity nanofillers emerges as a pivotal strategy. Nevertheless, the inherent chemical inertness of ideal nanofillers, such as boron nitride (BN), renders traditional modification strategies ineffective in reconciling the notorious “trade‐off” between thermal conductivity and electrical insulation. Herein, the fabrication of high‐performance EP composites utilizing BN nanofillers functionalized via a two‐step modification strategy involving KH550 and G‐POSS was reported through systematic physicochemical characterization and performance evaluation in this work. The underlying mechanisms governing the electro‐thermal regulation are elucidated, aiming to optimize the fabrication protocol for achieving superior comprehensive performance.

**FIGURE 2:**
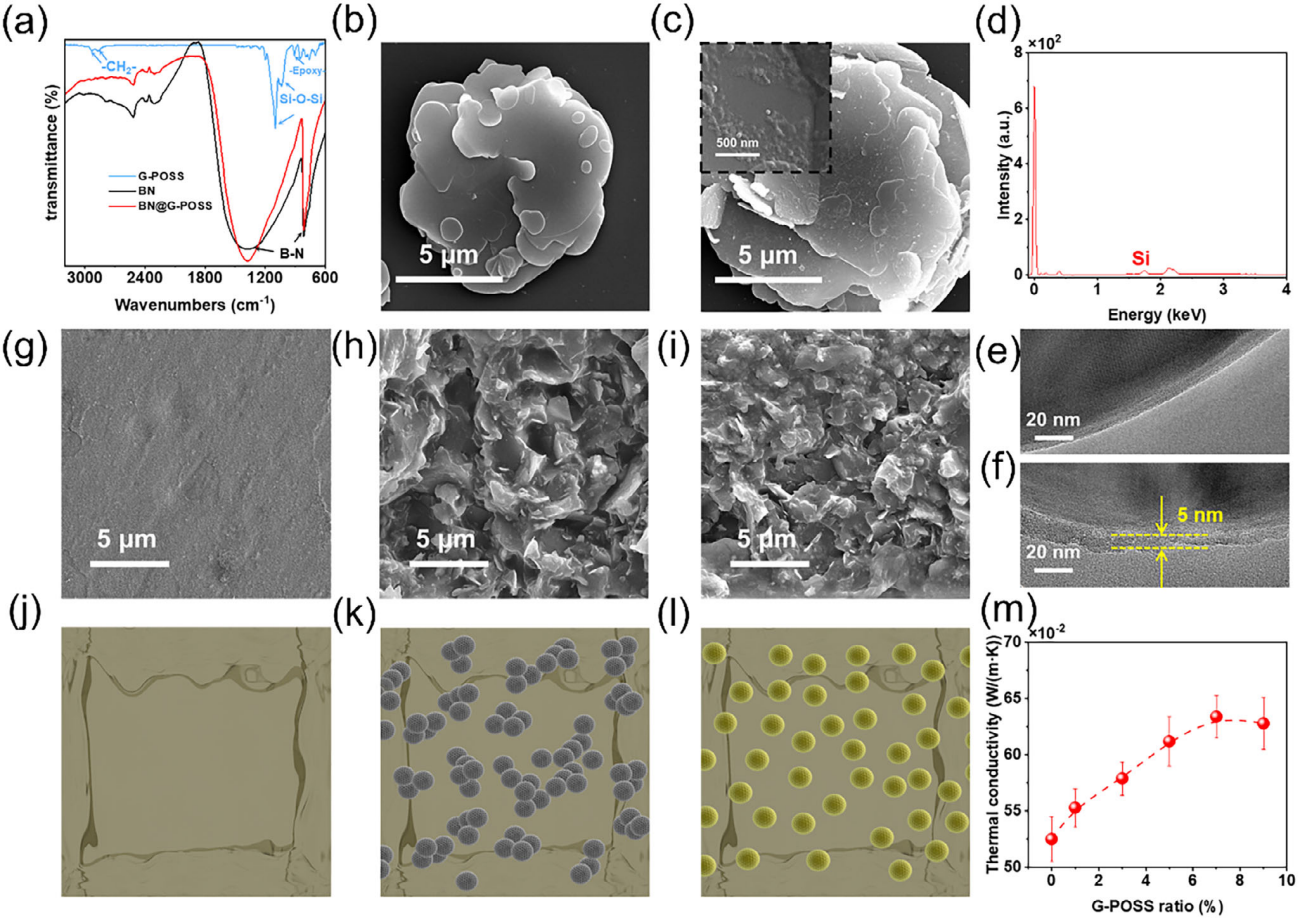
(a) FTIR spectra of BN, G‐POSS and BN@G‐POSS; SEM images of (b) pristine BN and (c) BN@G‐POSS; (d) EDS intensity distribution; TEM images of (e) pristine BN and (f) BN@G‐POSS; SEM micrographs of cross‐sectional morphology for (g) pristine EP, (h) BN/EP, and (i) BN@G‐POSS/EP; (j) Diagram of pristine EP; Proper distribution state of (k) pristine BN and (l) BN@G‐POSS in EP matrix; (m) Thermal conductivity of BN@G‐POSS under various G‐POSS concentrations.

Fourier‐transform infrared (FTIR) spectroscopy was employed to investigate the chemical structure of BN@G‐POSS, as shown in Figure 2a. Both pristine BN and BN@G‐POSS exhibit characteristic absorption bands at 817 and 1370 cm^−1^, which are attributed to the in‐plane B‐N stretching vibration and the out‐of‐plane B‐N‐B bending vibration, respectively [35]. In addition, pristine G‐POSS and BN@G‐POSS display pronounced peaks at 1100 and 1037 cm^−1^, corresponding to the asymmetric and symmetric stretching vibrations of Si‐O‐Si, respectively. The appearance of the ‐CH_2_‐ asymmetric and symmetric stretching vibrations at 2932 and 2869 cm^−1^ further confirms the successful grafting of G‐POSS onto the BN surface. Notably, the characteristic epoxy absorption band at 909 cm^−1^ is observed only in the pristine G‐POSS spectrum, while it disappears in the BN@G‐POSS sample [36]. This disappearance is attributed to the ring‐opening reaction of the epoxy groups during surface modification, providing strong evidence for the successful ring‐opening grafting of G‐POSS onto BN.

The surface morphologies of pristine BN and the BN@G‐POSS were examined by SEM. Pristine BN (Figure 2b) exhibits a smooth surface, whereas BN@G‐POSS (Figure 2c) shows a uniformly roughened surface after G‐POSS grafting, indicating a successful encapsulation. This is further supported by energy‐dispersive X‐ray spectroscopy (EDS) mapping, which reveals a homogeneous distribution of organic elements (Si and N) across the BN@G‐POSS surface (Figure 2d and Figure S2). Moreover, TEM analysis clearly reveals a smooth interface of pristine BN (Figure 2e). After modification process, G‐POSS shell with a thickness of 5 nm (Figure 2f) was observed, indicating a core‐shell structure after G‐POSS modification.

To assess the influence of G‐POSS modification on nanofiller dispersion, the cross‐sectional morphologies of pristine EP, 10 vol% BN/EP, and 10 vol% BN@G‐POSS/EP (containing 1 wt.% G‐POSS) composites were examined. As shown in Figure 2g, the pristine EP exhibited a smooth and uniform fracture surface, indicative of a dense and homogeneous matrix. Upon incorporation of unmodified BN (Figure 2h), the fracture surface became significantly rougher, accompanied by pronounced BN agglomerates, reflecting poor dispersion and weak interfacial compatibility between BN and the epoxy matrix. In contrast, the BN@G‐POSS/EP composite (Figure 2i) displayed a markedly finer and more homogeneous microstructure, in which large aggregates are effectively suppressed, demonstrating substantially improved nanofiller dispersion. To better illustrate the underlying dispersion states, schematic representations were provided. Figure 2j depicted the homogeneous epoxy matrix without nanofillers, Figure 2k illustrated the aggregation‐prone distribution of unmodified BN, and Figure 2l highlighted the uniform dispersion of BN@G‐POSS enabled by the organic‐inorganic hybrid G‐POSS shell. These schematics qualitatively captured the dispersion evolution observed in the SEM images and emphasized the role of G‐POSS modification in enhancing interfacial affinity and nanofiller distribution. The improved compatibility was further supported by sedimentation experiments conducted using pristine BN, KH550‐modified BN, and BN@G‐POSS in ethanol (Figure S3). After 30 min of settling, BN@G‐POSS remained well‐dispersed, whereas obvious sedimentation was observed for both BN and KH550‐BN, highlighting the superior dispersibility of G‐POSS@BN in organic solvent.

Considering these results, determining the optimal G‐POSS coating amount is crucial. Accordingly, the thermal conductivity of BN@G‐POSS/EP composites was systematically investigated as a function of the G‐POSS grafting ratio (0, 1, 3, 5, 7, and 9 wt.% relative to BN), while maintaining a constant nanofiller loading of 10 vol% to isolate the effect of grafting. As shown in Figure 2m, the thermal conductivity exhibited a distinct rise‐and‐fall trend, governed by the interplay between enhanced interfacial compatibility and increased interfacial thermal resistance. At grafting ratios below 7 wt.%, the thermal conductivity gradually increased, attributed to improved BN‐matrix compatibility and more uniform dispersion. These enhancements suppressed phonon scattering and facilitate the formation of continuous thermal pathways. The thermal conductivity reached a maximum of approximately 0.63 W m^−1^ K^−1^ at a grafting ratio of 7 wt.%, representing an optimal balance between interfacial bonding and heat‐transfer efficiency. When the grafting ratio exceeds this threshold, excessive G‐POSS encapsulation introduces additional interfacial thermal resistance and begins to disrupt effective heat conduction, leading to a decline in thermal conductivity [37]. Therefore, 7 wt.% was identified as the optimal grafting level for the subsequent fabrication of BN@G‐POSS/EP composites.

### 2.2 Dielectric and Thermal Properties

**FIGURE 3:**
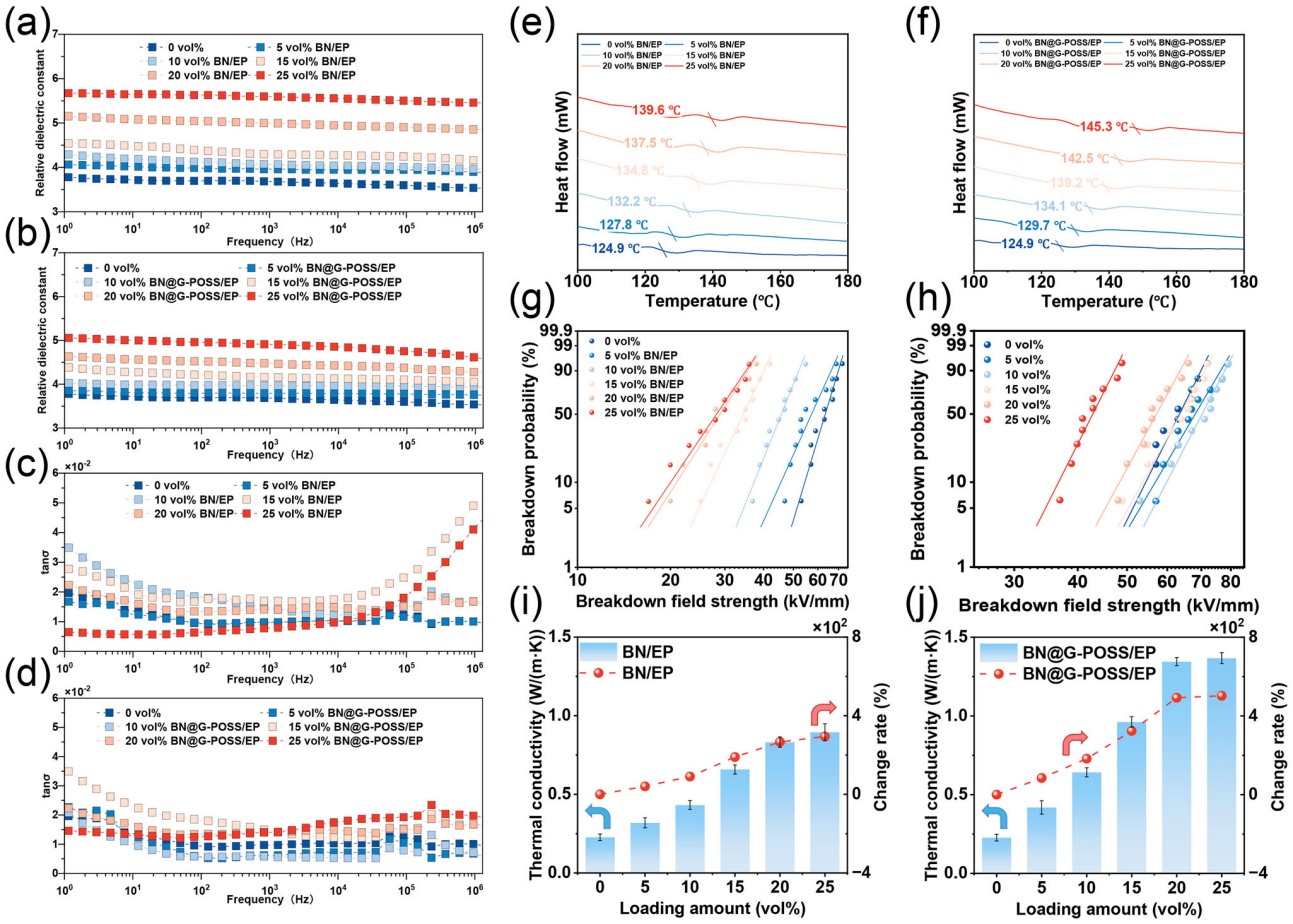
Relative dielectric constant of (a) BN/EP and (b) BN@G‐POSS/EP; Dielectric loss of (c) BN/EP and (d) BN@G‐POSS/EP; DSC curves of (e) BN/EP and (f) BN@G‐POSS/EP; Breakdown probability vs. breakdown field strength for (g) BN/EP and (h) BN@G‐POSS/EP at various loadings; Thermal conductivity and its change rate for (i) BN/EP and (j) BN@G‐POSS/EP under various voltages.

Dielectric properties are fundamental indicators for assessing the performance of electrical insulating materials [38]. The dielectric constant reflects the material's capacity for capacitive energy storage and owing to its pronounced frequency dependence, reveals the dynamic interactions between the internal microstructure and the external electric field. Meanwhile, dielectric loss characterizes the energy dissipated as heat under an applied field. Under combined high‐frequency, high‐d*v*/d*t*, and thermal stresses, dielectric loss tends to increase sharply, accelerating the degradation of epoxy insulation. To elucidate these effects, broadband dielectric spectroscopy was performed to evaluate the permittivity and dielectric loss of BN/EP and BN@G‐POSS/EP composites over a frequency range of 1–10^6^ Hz. As shown in Figure 3a,b, the relative dielectric constant (ε′) of both composites decreased with increasing frequency yet rises monotonically with higher nanofiller loading. At 100 kHz, ε′ of BN/EP (5–25 vol%) was 3.94, 4.01, 4.24, 4.91, and 5.50, respectively, whereas the corresponding BN@G‐POSS/EP composites exhibited lower values of 3.76, 3.94, 4.05, 4.28, and 4.61. Across all loadings, ε′ was consistently lower in BN@G‐POSS/EP than in BN/EP, reflecting the reduced interfacial polarization enabled by G‐POSS modification.

Figure 3c,d present the dielectric loss tangent (tanδ). BN/EP exhibited a marked increase in tanδ with both frequency and nanofiller loading, particularly in the high‐frequency range (10^4^–10^6^ Hz). By contrast, BN@G‐POSS/EP displayed a non‐monotonic behavior, in which tanδ first decreased and then increased with increasing loading, reaching a minimum at 10 vol%. At 10^5^ Hz, tanδ for BN@G‐POSS/EP (5–25 vol%) ranged from 0.007 to 0.019, substantially lower than the 0.020–0.059 measured for BN/EP. From an application standpoint, ε′ affects capacitance and signal propagation, while tanδ directly relates to heat generation and long‐term stability [39]. The consistently suppressed tanδ observed in BN@G‐POSS/EP demonstrated the effectiveness of G‐POSS in mitigating high‐frequency dielectric loss. The resulting combination of moderate ε′ and significantly reduced dielectric loss made BN@G‐POSS/EP as a promising candidate for high‐frequency and high‐stress insulation systems.

In addition to dielectric behavior, the glass transition temperature (Tg) provides essential guidance for optimizing curing conditions and assessing thermal reliability. Differential scanning calorimetry (DSC) results of BN/EP (Figure 3e) and BN@G‐POSS/EP (Figure 3f) revealed that Tg increased with nanofiller loading for both composite systems, with BN@G‐POSS/EP consistently achieving higher values than BN/EP. At 25 vol%, BN@G‐POSS/EP reached a Tg of 145.3°C, which was 5.7°C higher than that of BN/EP (139.6°C) and 20.4°C higher than pristine EP. A higher Tg ensured better retention of mechanical rigidity and dimensional stability under elevated temperatures, a crucial requirement for reliable operation under HFT overload conditions [40]. This Tg enhancement arises from two mechanisms: (1) hydrogen bonding between polar groups in G‐POSS and the epoxy network restricts polymer chain segmental motion [41], and (2) the reactive organic substituents in G‐POSS (e.g., epoxy and amine groups) form covalent bonds with epoxy resins or curing agents, thereby increasing cross‐link density and elevating Tg [42].

Since these composite materials are primarily intended for practical applications in AC (alternating current) electrical systems, testing their AC breakdown strength is crucial to accurately evaluate their insulation reliability under real‐world operating conditions. The AC breakdown performance of the composites was further evaluated using a two‐parameter Weibull statistical model (Figure S4). For BN/EP (Figure 3g), the breakdown strength exhibits a clear and continuous deterioration as the nanofiller loading increases from 5 to 25 vol%. The Weibull characteristic strength (E_0_) showed an evident leftward shift with higher BN content, and the scattering of data points broadens, indicating reduced reliability. This degradation arises from the intrinsic incompatibility and chemical inertness of pristine BN, which promote severe particle agglomeration within the epoxy matrix. The resulting interfacial voids and heterogeneous microstructure generated localized electric‐field intensification and weakly bonded regions that acted as preferential channels for charge injection and streamer initiation, ultimately accelerating electrical failure. In striking contrast, the BN@G‐POSS/EP composites (Figure 3h) displayed a markedly different trend. The breakdown strength initially increases from 0 to 10 vol% and remains relatively stable up to 20 vol%, accompanied by a noticeably narrower data distribution. This improvement stemmed from the enhanced interfacial adhesion and uniform dispersion imparted by the G‐POSS coating, which reduces defect density and establishes a more robust blocking interface against charge transport. Only at extremely high nanofiller loading (25 vol%), the breakdown strength began to decline, likely due to excessive viscosity during processing that hampers uniform dispersion and leaded to the reappearance of structural defects. Overall, the Weibull analysis demonstrated that while BN/EP suffers progressive deterioration in dielectric strength with increasing nanofiller loading, BN@G‐POSS/EP maintained high breakdown strength compared with BN/EP or POSS/EP (Table S1) and statistical reliability across a wide range of loadings. This underscored the critical role of G‐POSS in mitigating interfacial defects and enhancing the dielectric robustness of EP‐based insulation systems.

A comprehensive evaluation of dielectric, thermal, and processing characteristics clearly highlighted the superior performance of BN@G‐POSS/EP. As shown in Figure 3i,j, both BN/EP and BN@G‐POSS/EP exhibited a monotonic increase in thermal conductivity (λ) with increasing nanofiller loading, with an apparent percolation behavior beyond 10 vol%. However, at all loading levels, BN@G‐POSS/EP consistently outperforms BN/EP due to the improved interfacial coupling and homogeneous dispersion imparted by the G‐POSS coating. At 20 vol%, λ increased to approximately 1.15 W m^−1^·K^−1^ for BN@G‐POSS/EP, compared with 0.90 W m^−1^·K^−1^ for pristine BN/EP, representing a significantly enhanced growth rate. At 25 vol%, BN@G‐POSS/EP reached a maximum λ of 1.37 W m^−1^·K^−1^, again substantially surpassing its unmodified counterpart.

However, thermal conductivity cannot be considered in isolation. As demonstrated earlier, the AC breakdown strength deteriorated sharply at 25 vol% for both systems, indicating that excessive nanofiller content introduces structural defects and weakens electrical reliability. Furthermore, rheological measurements reinforced this limitation: To ensure a defect‐free casting process for HFT, specifically preventing the formation of bubbles and voids, the viscosity of the casting material must not exceed 4000 mPa·s [43]. At 100°C, the viscosities of 25 vol% BN/EP and BN@G‐POSS/EP reach 5152 and 4955 mPa·s, respectively, far exceeding the allowable range for defect‐free casting or potting. Such excessively high viscosity not only impaired nanofiller dispersion but also promoted void formation, which further limits breakdown strength and thermal transport pathways.

In contrast, the 20 vol% composites achieved a balanced and desirable property profile. They maintained thermal conductivities close to their respective maximum values while preserving a breakdown strength comparable to pristine EP. Importantly, their viscosities remained within a processable range, ensuring defect‐free molding and reliable structural integrity. Considering the differing sensitivities of thermal conduction, electrical insulation, and processability to nanofiller loading (Figure S5), 20 vol% was therefore identified as the optimal concentration. This loading achieved the best compromise among thermal management, dielectric robustness, and manufacturability, and is thus selected for all subsequent investigations.

### 2.3 High‐Frequency Partial Discharge Testing

EP encapsulation materials in HFTs are chronically subjected to intense coupled electrical‐thermal stresses, characterized by high operating frequencies, steep d*v*/d*t*, and elevated temperatures, all of which markedly accelerate insulation deterioration. Partial discharge acts both as the primary trigger and a key indicator of this degradation, typically originating from regions with strongly distorted electric fields or intrinsic defects within the insulation [44]. To emulate such rigorous operating conditions, a dedicated PD testing platform capable of applying coupled PET stresses was established (Figure S6).

**FIGURE 4:**
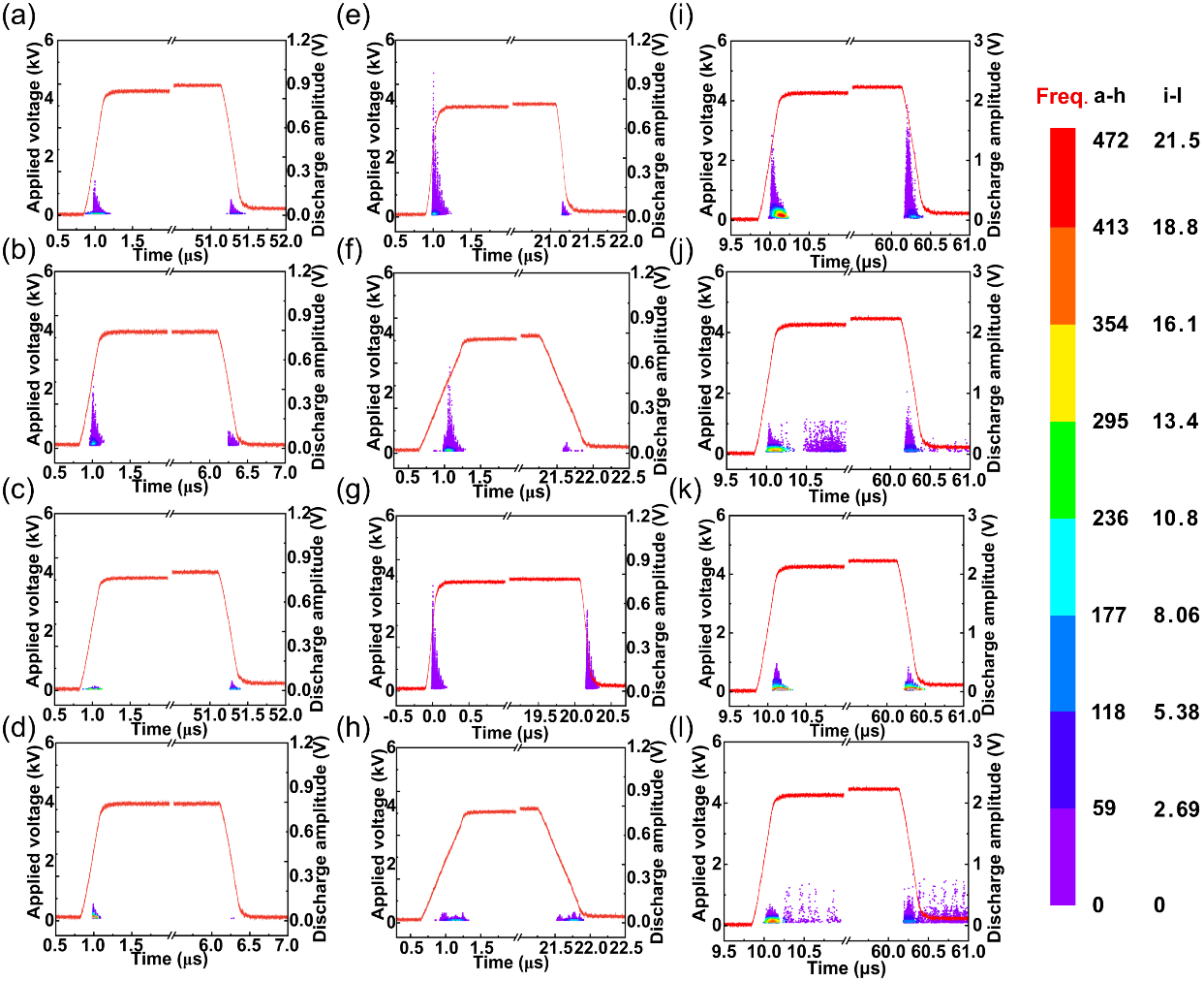
Partial discharge TRPD spectra of amplitude vs. time under the frequency of (a) 10 kHz and (b) 100 kHz for pristine EP; Partial discharge TRPD spectra of amplitude vs. time under the frequency of (c) 10 kHz and (d) 100 kHz for BN@G‐POSS/EP; Partial discharge TRPD spectra of amplitude vs. time under the rise time of (e) 100 ns and (f) 500 ns for pristine EP; Partial discharge TRPD spectra of amplitude vs. time under the rise time of (g) 100 ns and (h) 500 ns for BN@G‐POSS/EP; Partial discharge TRPD spectra of amplitude vs. time at (i) 20°C and (j) 140°C for pristine EP; Partial discharge TRPD spectra of amplitude vs. time at (k) 20°C and (l) 140°C for BN@G‐POSS/EP.

The time‐resolved partial discharge (TRPD) spectra of pristine EP and BN@G‐POSS/EP under various voltage frequencies were also presented. For consistent comparison, the time axis of the rising and falling edges of the applied waveform was normalized, and the rise/fall time was fixed at 200 ns with a 50% duty cycle. Both systems exhibited a characteristic triangular discharge pattern concentrated at the rising and falling flanks of the pulse voltage. As a key indicator of PD severity, the discharge amplitude of pristine EP reached 0.235 V at 10 kHz and 0.496 V at 100 kHz (Figure 4a,b), with discharge events broadly dispersed across the rising edge and dominated by high‐amplitude pulses. In stark contrast, BN@G‐POSS/EP (Figure 4c,d) displayed dramatically reduced amplitudes of only 0.0639 and 0.111 V at the same frequencies, accompanied by highly concentrated PD events. This transition from scattered to clustered PD suggested a higher discharge‐initiation threshold and significantly improved insulation robustness. Quantitative analysis (Figure S7) further confirmed this enhancement. Across 10–100 kHz, pristine EP exhibited maximum discharge amplitudes of 0.235, 0.891, 0.774, 0.455, and 0.496 V, whereas BN@G‐POSS/EP (Figure S8) showed markedly lower values of 0.0639, 0.206, 0.177, 0.372, and 0.111 V, with a maximum reduction of 0.685 V. Additionally, both PD pulse count and discharge count (Figure S9) were substantially suppressed. These findings demonstrated that under high‐frequency stress, BN@G‐POSS/EP effectively mitigated PD amplitude and activity, thereby providing superior insulation protection.

Compared with conventional Si devices, high‐voltage SiC wide‐bandgap power devices achieve switching speeds on the order of tens of nanoseconds, generating d*v*/d*t* stresses of several to tens of V ns^−1^ in PET systems. As shown in Figure 4e,f, pristine EP exhibited pronounced discharge amplitudes of 0.976 V at a 100‐ns rise time and 0.571 V at 500 ns. The discharge amplitude generally decreased with increasing rise time, while the PD events remain widely distributed across the rising edge, dominated by high‐amplitude pulses. By contrast, BN@G‐POSS/EP (Figure 4g,h) displayed significantly reduced amplitudes of 0.72 V (100 ns) and 0.071 V (500 ns), with discharge events highly concentrated. This shift indicated a markedly elevated discharge‐initiation threshold. A detailed comparison reveals maximum discharge amplitudes of 0.976, 0.891, 0.200, 0.688, and 0.571 V for pristine EP (Figure S10) across rise times of 100–500 ns, vs. 0.72, 0.206, 0.055, 0.118, and 0.071 V for BN@G‐POSS/EP (Figure S11). Again, the maximum reduction reaches 0.685 V, and PD pulse count and discharge count (Figure S12) were significantly suppressed. These results confirm that increasing the rise time further enhances the PD resistance of the modified material.

In practical HFT operation, compact geometry, high switching frequency, and dielectric heating‐induced losses can generate hotspot temperatures exceeding 160°C, far beyond the Tg of conventional potting encapsulants. Under such synergistic high‐temperature and high‐frequency stresses, the physicochemical properties, dielectric losses, and space‐charge behavior of EP drastically changed, thereby influencing its PD response. As shown in Figure 4i,j, pristine EP exhibited amplitudes of 1.925 V at 20°C and 1.02 V at 140°C. Although the amplitude generally decreased with temperature, the PD events became increasingly dispersed along the rising edge, with high‐amplitude pulses dominating at elevated temperatures. In contrast, BN@G‐POSS/EP (Figure 4k,l) showed amplitudes of only 0.416 V (20°C) and 0.83 V (140°C), maintaining a highly concentrated PD distribution even under severe thermal stress. This behavior revealed a substantially increased discharge‐initiation threshold and enhanced insulation integrity. Quantitative evaluation further substantiated these improvements. Across 20°C–140°C, pristine EP (Figure S13) exhibited maximum discharge amplitudes of 1.925, 3.402, 2.237, and 1.02 V, while BN@G‐POSS/EP (Figure S14 and Table S2) showed only 0.416, 1.598, 0.474, and 0.83 V, with a maximum differential of 1.804 V. Although PD amplitude and frequency typically intensify at elevated temperatures (particularly beyond 110°C), reflecting accelerated insulation aging, the BN@G‐POSS/EP composite consistently maintained suppressed PD activity. It can be observed that the BN@G‐POSS/EP composite exhibits a significantly decreased discharge counts compared to pristine EP (Figure S15). This clearly demonstrated that the incorporation of BN@G‐POSS effectively restrained PD evolution under coupled thermal‐electrical stress, substantially enhancing the operational reliability of HFT insulation.

### 2.4 Electrical Properties of the Materials

To further elucidate the mechanisms underlying the superior insulation performance of BN@G‐POSS/EP under coupled high‐frequency electrical and thermal stresses, a comprehensive analysis of its PD evolution was conducted. While the TRPD spectra revealed the suppression of discharge amplitude and pulse density under varying frequency, rise time, and temperature, these observations alone cannot fully clarify how the composite interacts with the transient electric field and space‐charge dynamics during fast‐switching PET operation. Therefore, correlating the measured PD characteristics with the intrinsic discharge mechanisms became essential for understanding how BN@G‐POSS modulates charge transport, discharge inception, and energy dissipation.

**FIGURE 5:**
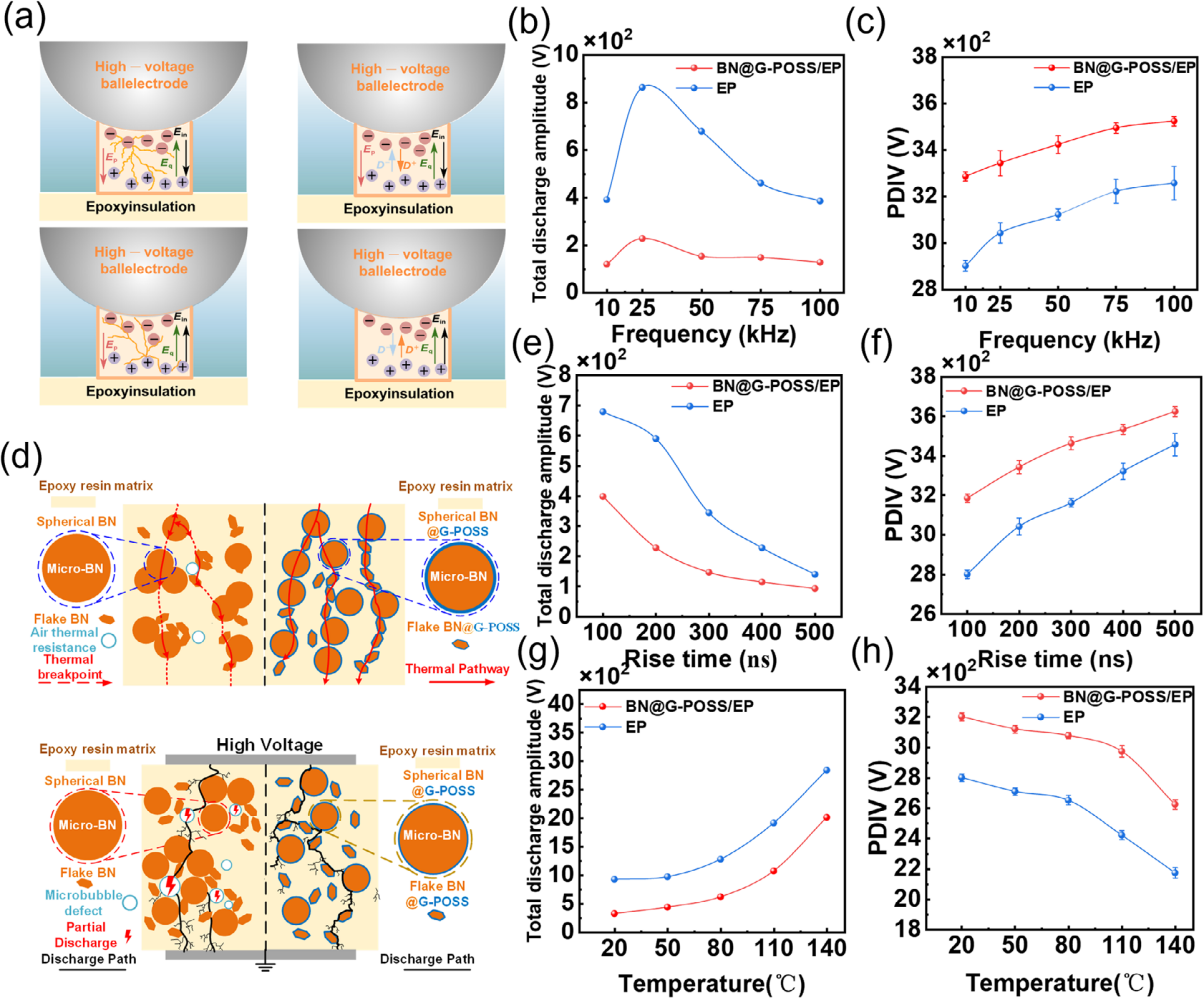
(a) Schematic of PD mechanism in high‐voltage spherical and epoxy insulation; (b) Total discharge amplitude and (c) PDIV distribution at 10–100 kHz Frequency of pristine EP and BN@G‐POSS/EP; (d) Schematic diagram of thermal and electrical conductivity pathways in BN/EP and BN@G‐POSS/EP; (e) Total discharge amplitude and (f) PDIV distribution at 100–500 ns rise time of pristine EP and BN@G‐POSS/EP; (g) Total discharge amplitude and (h) PDIV distribution at 20°C–140°C of pristine EP and BN@G‐POSS/EP.

Figure 5a illustrated the evolution of PD pulses under a typical high‐frequency voltage waveform. During the rising edge, the applied electric field (E_p_) gradually exceeded the ionization threshold of the air gap (E_ra_), initiating electron‐ion formation. Charge separation generated a space‐charge field (E_q_) opposite to E_p_, and discharge occurs once the combined field (E_in_) surpasses the inception threshold (E_th_). The rapid formation and collapse of conductive channels gave rise to repetitive PD pulses, whose impact progressively cleaved polymer chains and forms initial gas‐filled defects. In the high‐voltage plateau, excessive charge trapping enhances E_q_ to the extent that E_in_ was suppressed below E_th_, temporarily inhibiting PD. During the falling edge, the slow relaxation of space charge causes E_q_ to exceed E_p_, triggering reverse PD events until charge carriers were exhausted, after which the system returns to a discharge‐free state.

Figure 5b,c compared the frequency‐dependent PD characteristics of pristine EP and BN@G‐POSS/EP. For pristine EP, the total discharge amplitude first increased and then decreased with frequency, peaking at 25 kHz. In contrast, the composite maintained consistently lower amplitudes across all frequencies, indicating that BN@G‐POSS significantly suppresses discharge energy. The discharge repetition rate followed a similar trend. Although the PDIV of pristine EP exhibits only slight frequency dependence, BN@G‐POSS/EP showed a substantially higher PDIV throughout the tested range, demonstrating superior resistance to frequency‐induced discharge initiation.

The improved performance arises from synergistic thermal and electrical mechanisms enabled by the BN@G‐POSS architecture (Figure 5d). G‐POSS grafting enhanced the interfacial compatibility of BN, achieving more uniform dispersion and forming denser thermal conduction networks. Chemical bonding between modified BN and EP reduced phonon scattering, facilitating efficient heat transfer. Electrically, the introduction of G‐POSS facilitates the uniform dispersion of BN and mitigates agglomeration, thereby reducing voids and defects. This structural enhancement minimizes partial discharge and optimizes the discharge pathways, while the cage‐like POSS structure introduces deep traps that immobilize charge carriers and inhibit space‐charge accumulation [45]. These combined effects significantly enhance breakdown strength and PD resistance. When the nanofiller content exceeds 20 vol%, aggregation and void formation interrupted these networks, creating weak interfaces that reduce PD suppression.

Figure 5e,f show the dependence of PD behavior on voltage rise time. For both materials, the discharge amplitude decreased monotonically as the rise time increases from 100 to 500 ns, corresponding to a reduction in instantaneous electric‐field stress. Across all rise times, BN@G‐POSS/EP exhibited markedly lower discharge amplitudes and pulse counts. Meanwhile, the PDIV increased with rise time, and the composite consistently maintained a much higher threshold than pristine EP, confirming its stronger resistance to fast‐rising voltage stress.

The temperature‐dependent PD behavior is presented in Figure 5g,h. As temperature rises, the discharge amplitude of both systems increased sharply, particularly beyond 110°C, due to thermally enhanced carrier mobility and reduced polymer chain rigidity [39]. Despite this trend, BN@G‐POSS/EP consistently maintained significantly lower discharge amplitudes and repetition rates compared with pristine EP. While the PDIV of pristine EP dropped substantially with temperature, BN@G‐POSS/EP exhibited a slower decline and remains higher across the entire temperature range. This indicated that BN@G‐POSS effectively restrains thermally induced PD development by stabilizing charge transport processes and suppressing electrical‐tree propagation [46]. Overall, the BN@G‐POSS/EP composite demonstrated consistently lower discharge magnitudes, fewer discharge events, and higher inception voltages across variations in frequency, rise time, and temperature. These enhancements stemmed from improved filler‐matrix compatibility, deep‐trap‐assisted charge immobilization, and the formation of elongated discharge paths, collectively ensuring markedly improved PD resistance and insulation reliability for high‐frequency transformer applications. These findings confirmed that the BN@G‐POSS framework provided robust and stable PD suppression across a wide range of electrical and thermal stresses. By maintaining lower discharge activity and higher inception thresholds, the composite ensured more reliable insulation performance under fast‐switching and high‐temperature conditions, underscoring its strong potential for deployment in next‐generation HFT systems.

## 3 Conclusion

By integrating a rationally selected EP matrix, high‐thermal‐conductivity nanofillers, and a tailored KH550/G‐POSS surface‐grafting route, this work established a new encapsulation system specifically engineered for the coupled electrical‐thermal stresses encountered during fast‐switching PET operation. A dedicated PD evaluation platform was developed to capture the discharge behavior and degradation mechanisms under realistic pulse voltages, Through BN incorporation and controlled KH550/G‐POSS grafting, core‐shell BN@G‐POSS nanofillers with excellent interfacial compatibility and dispersion were successfully synthesized. The composite demonstrates pronounced improvements in thermal transport and dielectric integrity, achieving an optimal combination at 20 vol% nanofiller loading and 7 wt.% G‐POSS content relative to BN. The resulting BN@G‐POSS/EP exhibits a high thermal conductivity of 1.35 W m^−1^ K^−1^ (492.5% higher than pristine EP) with only a modest 9.6% reduction in AC breakdown strength. Under identical pulse excitation (10 kHz, 100 ns rise time), the PD inception voltage surpasses that of BN/EP by 14.3%–22.7% across 20°C–110°C, reflecting significant improvements in high‐frequency insulation capability. Overall, this work resolves the long‐standing trade‐off between thermal conductivity and high‐frequency PD resistance by enabling simultaneous heat‐dissipation enhancement and discharge suppression. The proposed BN@G‐POSS/EP composite, together with the integrated materials‐device co‐optimization methodology, provides a robust foundation for designing long‐lifetime, high‐reliability encapsulation materials for next‐generation high‐frequency transformers and advanced power electronic systems.

## 4 Experimental Section

### 4.1 Preparation of BN@G‐POSS Nanofiller

The whole preparation process of BN@G‐POSS can be referenced in Figure S16. A mixed solvent of anhydrous ethanol and deionized water (9:1, v/v) was prepared, and the pH was adjusted to strongly basic environment using aqueous ammonia. KH550 (2–5 wt.%, relative to the mass of BN) was then slowly added dropwise to the solution under magnetic stirring at room temperature. The hydrolysis reaction was allowed to proceed for 30 min to ensure the complete conversion of ethoxy groups into silanol groups.

BN powder was first dried in a vacuum oven at 60°C for 12 h and subsequently refluxed in concentrated nitric acid (65 wt.%) at 80°C for 6 h to introduce surface hydroxyl groups. After thorough washing with deionized water until neutral pH and drying at 60°C, the hydroxylated BN was dispersed into the hydrolyzed KH550 solution at a BN:KH550 mass ratio of 1:0.1–1:0.3. The mixture was sonicated for 30 min to achieve uniform dispersion and then stirred in a water bath at 60°C for 4–6 h to promote the condensation reaction between silanol groups and the hydroxylated BN surface. The resulting KH550‐functionalized BN was collected by centrifugation, washed repeatedly with anhydrous ethanol, and dried in a forced‐air oven at 60°C for 12 h.

G‐POSS was first dissolved in anhydrous tetrahydrofuran (THF) at a concentration of 5–10 wt.% and sonicated for 30 min to obtain a homogeneous solution, followed by nitrogen purging for 10 min to remove dissolved oxygen. Under a nitrogen atmosphere, the dried KH550‐functionalized BN was dispersed in anhydrous THF at a solid‐liquid mass ratio of 1:20 and sonicated for 30 min. Subsequently, the G‐POSS/THF solution was slowly added dropwise to the KH550‐BN dispersion, with the G‐POSS content controlled at 1, 3, 5, 7, and 9 wt.% relative to the mass of KH550‐BN. Triethylamine, in an amount equal to the mass of G‐POSS, was then introduced as an acid scavenger. The reaction was carried out under magnetic stirring in an oil bath at 60°C–80°C for 24 h under a nitrogen atmosphere and protected from light. After completion, the resulting product was collected by centrifugation and washed three times with THF to remove unreacted G‐POSS, followed by three additional washes with anhydrous ethanol to replace the residual THF. Finally, the BN@G‐POSS product was dried in a vacuum oven at 60°C for 24 h.

### 4.2 Preparation of Pristine EP

Bisphenol A‐type epoxy resin (50 g) was first preheated in a blast oven at 50°C to remove residual moisture and volatile components and to reduce its viscosity. The preheated resin was then blended with cycloaliphatic epoxy at a mass ratio of 1:1 under continuous stirring until a homogeneous mixture was obtained. Subsequently, methylhexahydrophthalic anhydride (90 g) was added as the curing agent and stirred for 1 h, followed by the introduction of 2, 4, 6‐tri(dimethylaminomethyl)phenol (0.5 g) as the accelerator and further stirring for 30 min. The resulting mixture was degassed under vacuum at 50°C for 30 min with repeated evacuation‐venting cycles to eliminate trapped air bubbles and then allowed to stand for 10 min. Finally, the degassed resin was tape‐cast onto a polyethylene terephthalate (PET) release film using a preheated doctor‐blade apparatus at 60°C and cured using a stepped thermal program of 95°C for 30 min, 130°C for 2 h, and 155°C for 1 h. After cooling to room temperature, the cured EP films were demolded and cut into the desired dimensions for subsequent characterization.

### 4.3 Preparation of BN/EP and BN@G‐POSS/EP Composites

BN/EP and BN@G‐POSS/EP composites were fabricated following an identical protocol, with the nanofiller loading systematically varied at 5, 10, 15, 20, and 25 vol% relative to the total volume of the BN/EP mixture, while maintaining a constant total EP mass, in order to evaluate the effect of nanofiller content on the electro‐thermal properties (Figure S17). The preparation of the 10 vol% BN@G‐POSS/EP composite is described here as a representative example (Figure S18). Bisphenol A‐type epoxy resin was preheated at 50°C and blended with cycloaliphatic epoxy at a mass ratio of 1:1, after which dried BN or BN@G‐POSS powder (40.3 g) was gradually incorporated into the epoxy mixture. The system was subjected to ultrasonication in a water bath at 50°C for 30 min to promote filler wetting and initial dispersion, followed by mechanical stirring at 50°C and 2000 rpm for 2 h to achieve homogeneous distribution. Subsequently, methylhexahydrophthalic anhydride (90 g) was added and stirred for 1 h, and 2, 4, 6‐tri(dimethylaminomethyl)phenol (0.5 g) was introduced as the accelerator with an additional 30 min of stirring. The resulting mixture was degassed under vacuum at 50°C for 30 min with repeated evacuation‐venting cycles until all visible air bubbles were removed and then allowed to rest for 10 min. The degassed mixture was tape‐cast onto a PET release film using a preheated doctor‐blade apparatus at 60°C, with the film thickness controlled by adjusting the blade height. The cast films were cured using the same stepped thermal schedule as pristine EP (95°C for 30 min, 130°C for 2 h, and 155°C for 1 h), with extended post‐curing temperature and duration applied for composites with higher filler loadings. After cooling to room temperature, the cured composite films were demolded and cut into the required dimensions for subsequent testing and characterization.

## Author Contributions

**Yuan Wang**: conceptualization, investigation, funding acquisition, validation. **Dezhi Cui**: methodology, validation, writing – original draft. **Shuai Jiang**: conceptualization, writing – review and editing, writing – original draft, validation, formal analysis. **Shaojie Luo**: methodology, data curation, formal analysis, validation, writing – original draft, visualization, writing – review and editing. **Renli Fu**: investigation, formal analysis. **Jun Jiang**: conceptualization, data curation, supervision, writing – original draft, writing – review and editing, validation. **Zeya Huang**: validation, investigation, conceptualization, writing – review and editing.

## Conflicts of Interest

The authors declare no conflicts of interest

## Supporting information




**Supporting File**: advs75952‐sup‐0001‐SuppMat.docx.

## Data Availability

The data that support the findings of this study are available from the corresponding author upon reasonable request.
