# Supplementary material for: Synergistic Enhancement of Electrical‐Thermal Properties in KH550/G‐POSS Functionalized BN/Epoxy Composites for High‐Frequency Transformer Insulation
Source: Adv Sci (Weinh). 2026 Jun 2:e75952. Online ahead of print. doi: 10.1002/advs.75952 (PMC13336849; doi:10.1002/advs.75952)
Supplement: Supplementary file 1 — Supporting file: advs75952‐sup‐0001‐SuppMat.docx [file ADVS-9999-e75952-s001.docx]

*Supporting Information*

**Synergistic enhancement of electrical-thermal properties in G-POSS modified BN/Epoxy composites for high-frequency transformer insulation**

Jun Jiang ^1, *, #^, Shaojie Luo ^1, #^, Dezhi Cui ^2^, Zeya Huang ^3^, Shuai Jiang ^4, *^, Renli Fu ^3^, Yuan Wang ^5^

1. Department of Electrical Engineering, College of Automation Engineering, Nanjing University of Aeronautics and Astronautics, Nanjing 211106, China
2. Liaocheng Power Supply Company, State Grid Shandong Electric Power Company, Shandong 252000, China
3. College of Materials Science and Technology, Nanjing University of Aeronautics and Astronautics, Nanjing 210016, China
4. Research Center for Membrane and Film Technology, Department of Chemical Science and Engineering, Kobe University, Kobe 657-8501, Japan
5. School of Mechanics and Engineering Science, Peking University, Beijing 100871, China

# They contributed equally to this work.

* Corresponding author. E-mail: jiangshuai1213@163.com (S. Jiang); jiangjun0628@163.com (J. Jiang)

**Supplementary notes:**

***Materials***

*γ*-Aminopropyltriethoxysilane (KH550), Bisphenol A-type EP resin (grade E-51), and methylhexahydrophthalic anhydride (MHHPA) used in this study were purchased from Shanghai Macklin Biochemical Co., Ltd. (Shanghai, China). Spherical Boron Nitride (BN, 40 μm, 99%) was supplied by Ya'an Bestry High Performance Materials Co., Ltd. (Ya'an, China). Cycloaliphatic epoxy resin (CELLOXIDE 2021P) was obtained from Nanjing Qiangshan New Materials Co., Ltd. (Nanjing, China). 2,4,6-tri (dimethylaminomethyl)phenol (DMP-30) and Ethanol absolute were purchased from Sinopharm Chemical Reagent Co., Ltd. (Shanghai, China). Concentrated nitric acid (HNO_3_, 65%) and aqueous ammonia (NH_3_) were provided by Nanjing Chemical Reagent Co., Ltd. (Nanjing, China). Tetrahydrofuran (THF) and Triethylamine (TEA) were supplied by Shanghai Aladdin Biochemical Technology Co., Ltd. (Shanghai, China). All reagents used in this work were of analytical grade (purity > 99.7%).

***Characterization***

To validate the successful modification of BN by G-POSS and explore the optimal modification ratio, the modified chemical structure was obtained using a Thermo Fisher 616 FT-IR spectrometer. Surface morphology was examined using a field emission scanning electron microscope (SEM) model TESCAN LYRA3 GM from TESCAN BRNO, Czech Republic. The instrument’s integrated energy-dispersive X-ray spectrometer (EDS) performed area scanning to analyze the chemical element distribution corresponding to the SEM images. Microstructures were observed using a Thermo Fisher Talos F200s transmission electron microscope (TEM). For the thermal conductivity measurements, the prepared specimens were circular films with a diameter of 12.5 mm and a thickness ranging from 0.05 to 0.15 mm. Each specimen was tested in triplicate with a 15-minute interval between consecutive measurements to ensure thermal equilibrium, and the average values were reported. All tests were conducted at an ambient temperature of 25 °C. Thermal conductivity measurements were performed with a TPS 2500 S thermal conductivity tester from Hot Disk, Sweden.

The PD testing system was equipped with a UHF sensor (ZCCGQ-U-PZ-01, Beijing Huadian Zhicheng Electrical Equipment Co., Ltd., Beijing, China), a high-voltage probe (P6015A, Tektronix Inc., Beaverton, OR, USA), a high-frequency high-voltage pulse power supply (HVP-20P, Xi'an Lingfengyuan Electronic Technology Co., Ltd., Xi'an, China), and a digital storage oscilloscope (DS7014, RIGOL Technologies Co., Ltd., Suzhou, China).

For sample preparation and synthesis, the following instruments were employed: an electric thermostatic blast drying oven (DHG-9070A, Shanghai Yiheng Scientific Instrument Co., Ltd., Shanghai, China), an ultrasonic cleaner (KQ-50, Kunshan Ultrasonic Instrument Co., Ltd., Kunshan, China), and an electronic analytical balance (FA2204N, Shanghai Qinghai Instrument Co., Ltd., Shanghai, China). Solution processing involved a pH meter (PHS-2F, Shanghai Leici Instrument Factory, Shanghai, China), a collector constant temperature magnetic stirrer (DF-101S, Gongyi Yuhua Instrument Co., Ltd., Gongyi, China), a mechanical stirrer (JJ-1A, Changzhou Ronghua Instrument Manufacturing Co., Ltd., Changzhou, China), and a digital display heating stage (ET-1515, Shenzhen Bangqi Chuangyuan Technology Co., Ltd., Shenzhen, China). Additionally, a high-speed centrifuge (TG-16w, Xiangtan Xiangyi Instrument Co., Ltd., Xiangtan, China) and a vacuum pump (FY-1H-N, Zhejiang Value Mechanical & Electrical Products Co., Ltd., Wenling, China) were utilized for separation and degassing processes.

***Testing conditions***

Based on the Dinger-Funk particle packing theory, when particles of different sizes satisfy a specific distribution pattern, the voids between the fillers are minimized and the efficiency of establishing thermal conduction pathways is maximized. The ideal model using the following equation;

$U\left(D_{\text{p}}\right)=\frac{D_{\text{p}}^{\text{n}}-D_{\min}^{\text{n}}}{D_{\max}^{\text{n}}-D_{\min}^{\text{n}}}$ (1)

Where *U*(*D*_p_) represents the cumulative percentage of fillers with particle sizes smaller than *D*_p_; *D*_p_ denotes the particle size of the filler in micrometres (μm); *D*_min_ represents the minimum particle size of fillers in the composite system in micrometres (μm); *D*_max_ denotes the maximum particle size of fillers in the composite system in micrometres (μm); *n*is the distribution modulus, which is 0.37 for densest packing.

For composite systems with multi-scale particle sizes, the actual cumulative distribution satisfies;

$U'(D_{\text{p}})=\sum_{i=1}^{m} a_{\text{i}}U_{\text{Di}}$ (2)

where *U*′(*D*_p_) denotes the cumulative percentage of fillers with particle size less than *D*_p_; *a*_i_ represents the volume fraction of the *I* particle size filler relative to the total filler volume, where *i* is a positive integer; *U*_Di_ is the cumulative distribution function of the *i* particle size filler. The glass transition temperature (*T*_g_) provides an indication of a material's thermal resistance and may be employed to guide optimisation of curing processes. This chapter utilises a TA-Q200 Differential Scanning Calorimeter (DSC) from Thermal Analysis Instruments to analyse the thermal flow characteristics and thermal behavior of *T*_g_ of EP composite materials. During testing, 6–8 mg of sample was placed in an aluminium crucible and sealed. Under a nitrogen atmosphere, DSC curves were recorded at a constant heating rate of 10 °C/min from 25 to 180 °C. To ensure data accuracy, each sample was tested three times after the heating ramp had eliminated thermal history effects.

During AC breakdown testing, the prepared film specimens were cut into 40 × 40 mm squares, and the thickness of each specimen was individually measured using a digital vernier caliper. In accordance with the GB/T 1408.1-2016 standard, the voltage was applied at a constant ramp rate of 1 kV/s until electrical breakdown occurred. The breakdown voltage of each specimen was recorded, and this testing procedure was repeated for a total of 10 specimens to ensure statistical reliability.

During partial discharge (PD) testing, a high-frequency, high-voltage pulse power supply is connected to the partial discharge model via high-voltage cables to apply high-frequency pulse voltage stress to the insulating specimen. All power supply output waveform parameters are configured via the host computer control panel, whilst the plate electrodes are earthed. Both side walls of the electrically heated forced-air heating chamber feature openings for introducing the high-voltage cable and earth cable respectively. The internal temperature is regulated via the temperature control panel to apply thermal stress to the insulating specimen. The high-voltage probe attenuates the power supply voltage by a factor of 1000 before connecting to an oscilloscope for capturing the excitation voltage signal. The UHF antenna is positioned externally on the temperature-controlled chamber near the observation window to collect electromagnetic radiation signals generated by partial discharge. The oscilloscope enables real-time observation and recording of partial discharge phenomena. When clusters of signals exhibiting PD characteristics appear, PD is deemed to have occurred. A custom-developed programme facilitates real-time acquisition and storage of oscilloscope data for subsequent processing and analysis of the PD signals.

Unmodified and 20 wt% G-POSS-BN/EP composite materials served as test specimens. Specimens of 0.2 mm thickness were prepared via cast-film process and cut into 40 mm × 40 mm squares. To ensure thickness consistency, precision electronic vernier calipers were employed to screen cut specimens, with tolerances maintained below 0.02 mm. In accordance with IEC 60243-1, prior to partial discharge testing, the specimen surfaces were wiped with anhydrous ethanol. Specimens were then placed in a 60°C electric hot-air drying oven for 24 hours to remove surface contaminants and moisture.

***Partial discharge initiation voltage***

When the applied voltage or electric field strength reaches a certain value, the electric field strength at local defects within the insulating material satisfies the conditions for initiating discharge. This ionizes the air and generates free electrons, at which point PD occurs. This applied voltage is termed the PDIV. The PDIV is closely related to the properties of the insulating material, its thickness, the presence or absence of internal defects, and the voltage waveform and parameters. In accordance with IEC 60034-18-42, the PDIV of unmodified and modified EP specimens is tested using the uniform voltage-increase method at a rate of 0.2 kV/s to roughly estimate the initial PDIV range. The 70% value of the initial PDIV is selected as the voltage reference, with voltage increments set at 50 V to precisely determine the PDIV value. The PDIV measurement and determination process is illustrated in Figure 3.6. Voltage application ceases upon observing the first PD signal. Testing resumes only if no stable discharge occurs within the subsequent 30 seconds; otherwise, the PDIV value recorded at that point is retained. Given the typically random nature of PDIV, to ensure the universality of the PDIV test methodology, after each PDIV test, a specimen of identical process and thickness is substituted. Seven cyclic tests are conducted at each frequency, with the average value subsequently calculated.

***Time-resolved partial discharge spectra***

As a core diagnostic technique for assessing insulation condition, PD spectra reflect the temporal characteristics of discharge events, providing quantitative evidence for evaluating insulation degradation in electrical equipment. Within an observation window of equal duration, PD signals stimulated by high-frequency voltage exhibit greater density and pronounced pulse overlap effects compared to those stimulated by power-frequency voltage. Compared to Phase Resolved Partial Discharge (PRPD) spectra, Time Resolved Partial Discharge (TRPD) spectra possess sub-picosecond temporal resolution. This enables effective analysis of nanosecond-scale discharge pulse distribution variations, offering greater clarity in analyzing discharge activity and identifying discharge patterns. Consequently, TRPD spectra are more suitable for PD analysis under high-frequency pulse voltage conditions. By conducting multidimensional feature analysis of repetitive discharge events within the TRPD spectrum, including statistical parameters such as discharge shape, amplitude, timing, and frequency one can uncover the influence patterns of external parameter variations (e.g., voltage waveform, voltage parameters, temperature conditions) on the intrinsic evolutionary mechanisms of PD behavior in insulating media.

PD events exhibit pronounced statistical dispersion characteristics. To effectively mitigate the impact of discharge randomness on analysis and enhance TRPD spectrum visualization, this study continuously collected discharge pulse data from 50 single-cycle groups. Through noise signal filtering, discharge pulse extraction, and statistical analysis of characteristic parameters, TRPD spectra were plotted. Characteristic parameters including total discharge amplitude, cumulative discharge amplitude, discharge count, discharge repetition rate, and discharge delay time were employed to quantify PD characteristics. Mechanism of voltage parameters affecting PD .The spatial charge behavior at air gaps, along with frequency-dependent dielectric constants and dielectric loss factors, are the primary factors influencing PD.

***Spatial charge behavior***

The initial generation of electrons is primarily attributed to surface emission from trapped spatial charges within the solid insulating medium, exhibiting a degree of randomness. This randomness is one of the causes for the stochastic occurrence of discharges. The electron emission probability *λ*(t) approximately follows the Richardson-Schottky law:

$\lambda(t)=N_{\text{q}}(t)v_{0}\exp(-\frac{\psi-\sqrt{e\left|E(t)/4\pi\varepsilon_{0}\right|}}{KT})$ (3)

where *N*_q_(t)denotes the number of electrons used for escape at *t* time ; *ψ*(t) is the escape work function of the charge; *E*(t) is the composite electric field strength at the air gap at *t* time; *v*_0_ is the photoionization constant; *e* is the charge quantity; ε_0_ is the vacuum permittivity; *K* is the Boltzmann constant; and *T* is the Kelvin temperature.

From equation (3), it is evident that the larger *N*_q_(t) is, the higher *λ*(t) becomes, and the shorter the discharge delay time *t*_d_. Consequently, *t*_d_ and *N*_q_(t)are fundamentally inversely proportional, allowing the equation to be rewritten as:

$A_{\text{PD}}=c_{2}(V_{\min}-V_{\text{res}}+\frac{c_{4}k}{N_{\text{q}}(t)})$ (4)

where *c*_2_ and *c*_4_ are proportionality coefficients.

Through the above formula, the influence of voltage parameters on discharge characteristics is transformed into their effect on space-charge behavior. Both the discharge amplitude *A*_PD_ and discharge frequency *N*_PD_ decrease as *N*_q_(t) increases. The random nature of discharge occurrence is described as;

$P\left(t\right)=1-\exp(-\lambda N_{\text{e}}(t))$ (5)

where *P*(t) denotes the discharge probability at *t* time.

Furthermore, the spatial charge decay time constant correlates with charge drift or recombination rates, exhibiting decay times of approximately 2-1000 ms or even longer. This decay duration significantly exceeds the pulse duration under high-frequency pulsed voltages. As the rise time increases, discharge pulses become more dispersed in their temporal distribution. This has little effect on the dissipation of space charge, yet the accumulation rate increases significantly. Consequently, the weakening effect of the composite electric field is amplified, leading to a reduction in discharge amplitude. Regarding frequency, as frequency increases, the time required for space charge dissipation decreases. Charge continuously accumulates, greatly weakening the composite electric field. Thus, both discharge amplitude and discharge frequency decrease.

***Dielectric constant***

When high-frequency pulse excitation *V*_p_ is applied, the induced electric field in the air gap *E*_0_ is:

$E_{0}=\frac{E_{\text{p}}d_{0}}{d_{0}+(\frac{\varepsilon_{\text{r}}}{\varepsilon_{1}})d_{1}}$ (6)

where *d*_0_ and *d*_1_ denote the thicknesses of the air gap and insulating layer respectively; *ε*_r_ and *ε*_0_ represent the dielectric constants of the air gap and insulating layer respectively. As the dielectric constant of the air gap is lower than that of the solid medium, and *d*_0_ is relatively small compared to *d*_1_, the air gap experiences an electric field exceeding the dielectric constant ratio. This can be described by the field enhancement factor *f*_E_:

$f_{\text{E}}=\frac{\varepsilon_{\text{r}}(f)}{1+2\varepsilon_{\text{r}}(f)}$ (7)

where *f* is the frequency of the applied voltage. Considering the weakening effect of *E*_q_, equation (7) is rewritten as:

$E_{\text{in}}=\frac{\varepsilon_{\text{r}}(f)E_{\text{p}}}{1+2\varepsilon_{\text{r}}(f)}-E_{\text{q}}$ (8)

As frequency increases, the dielectric constant of the insulation gradually decreases. Consequently, establishing the same *E*_in_ requires a higher applied electric field to reach the *E*_th_ PD threshold. Furthermore, increased frequency shortens the period per cycle, reducing the time available for space charge accumulation. This diminishes the *E*_q_ damping effect of *E*_in_ on, making discharge more likely to occur.

***Dielectric loss factor***

Under high-frequency pulsed voltage, the dielectric loss per unit volume of solid insulating material is given by;

$\left\{\begin{matrix} p=(\gamma+g)E^{2} \\ g=\frac{\varepsilon_{0}(\varepsilon_{\text{s}}-\varepsilon_{\infty})\omega^{2}\tau}{1+\omega^{2}\tau^{2}} \end{matrix}\right.$ (9)

where *γ* is the volume conductivity of the insulating medium; *g* is the equivalent conductivity for relaxation polarization loss; *E* is the electric field strength; *ω* is the angular frequency of the applied voltage; *τ* is the relaxation time constant of the relaxation polarization process; *ε*_0_ is the vacuum permittivity; *ε*_s_ and *ε*_∞_ are the static and optical frequency permittivities of the material, respectively.

Thus, it follows from the above equation that dielectric loss increases with rising frequency. The combined impact of dielectric loss and discharge energy causes material temperature to rise, thereby increasing volumetric conductivity and further promoting heat generation within the material. Since dielectric loss and material temperature do not increase indefinitely with frequency, but rather reach equilibrium when frequency attains a certain value, with temperature stabilizing thereafter, this constitutes the primary cause of the frequency-induced inflection point phenomenon.

Although increasing frequency initially boosts both the total discharge amplitude and discharge frequency within a single cycle, the subsequent rise in cycles per unit time ensures that discharge repetition rate and cumulative discharge amplitude still increase. Consequently, insulation degradation intensifies with frequency over an identical timeframe. Beyond the thermal effects induced by frequency, the shortening of charge dissipation time with increasing frequency means that spatially accumulated charge from prior discharges fails to dissipate fully. Consequently, this accumulated charge inhibits subsequent discharges.


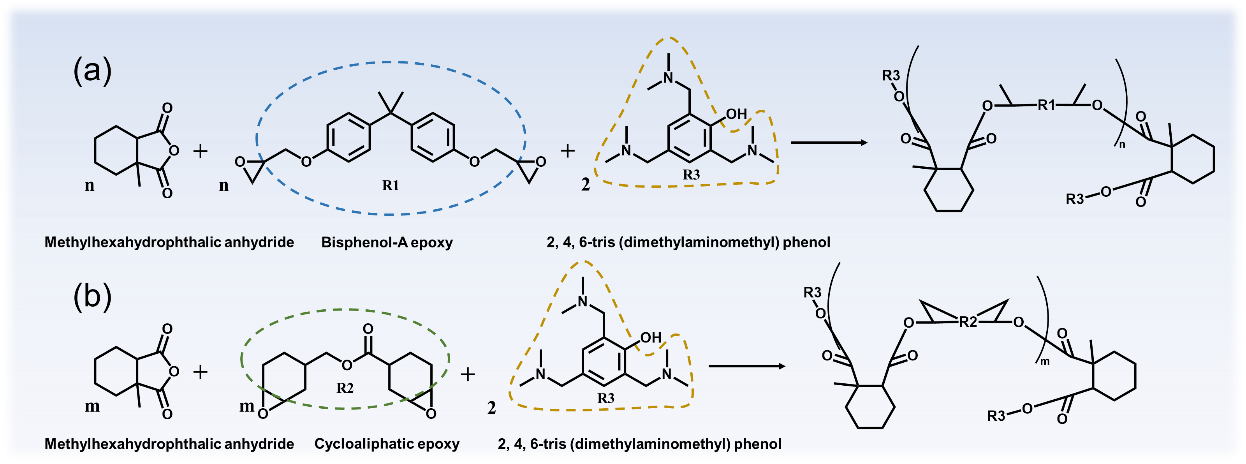


**Figure S1** Crosslinking reactions of (a) bisphenol A-type EP and (b) cycloaliphatic epoxy systems. (Methylhexahydrophthalic anhydride and 2, 4, 6-tri(dimethylaminomethyl) phenol were used as curing agent and initiator respectively).


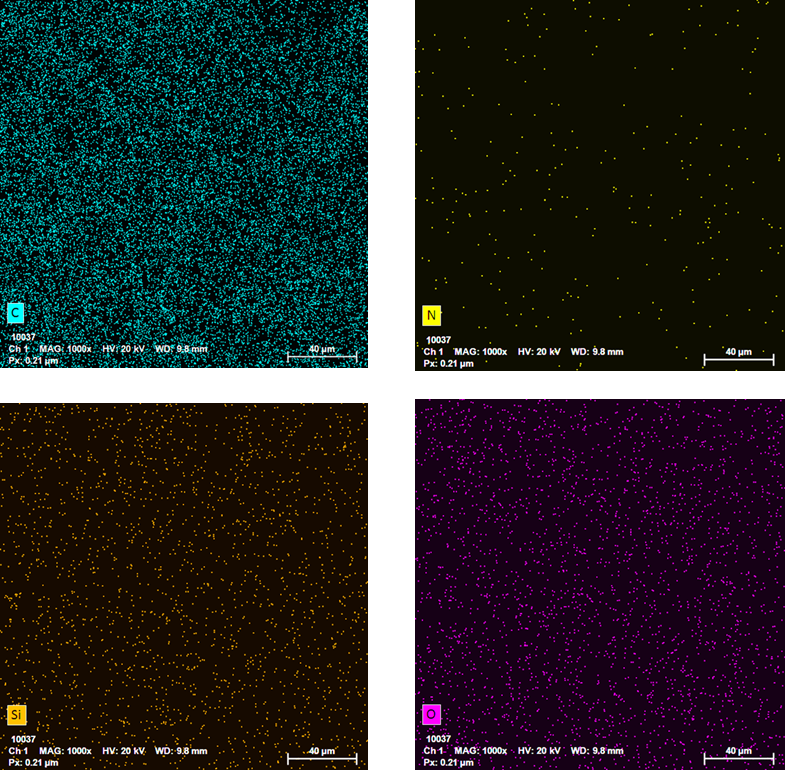


**Figure S2** Element mapping of BN@G-POSS composite.

**Figure S3** Sedimentation state of pure BN, KH550-BN and BN@G-POSS in ethanol. Specifically, 1 g each of BN, BN-KH550, and BN@G-POSS (7 wt.%) were added to absolute ethanol to prepare mixed suspensions of the same volume (82 mL). After ultrasonic dispersion for 10 min, their sedimentation rates within 1 h were measured.

**Figure S4** Diagram of the devices for breakdown test (HVAC: High voltage alternating current).


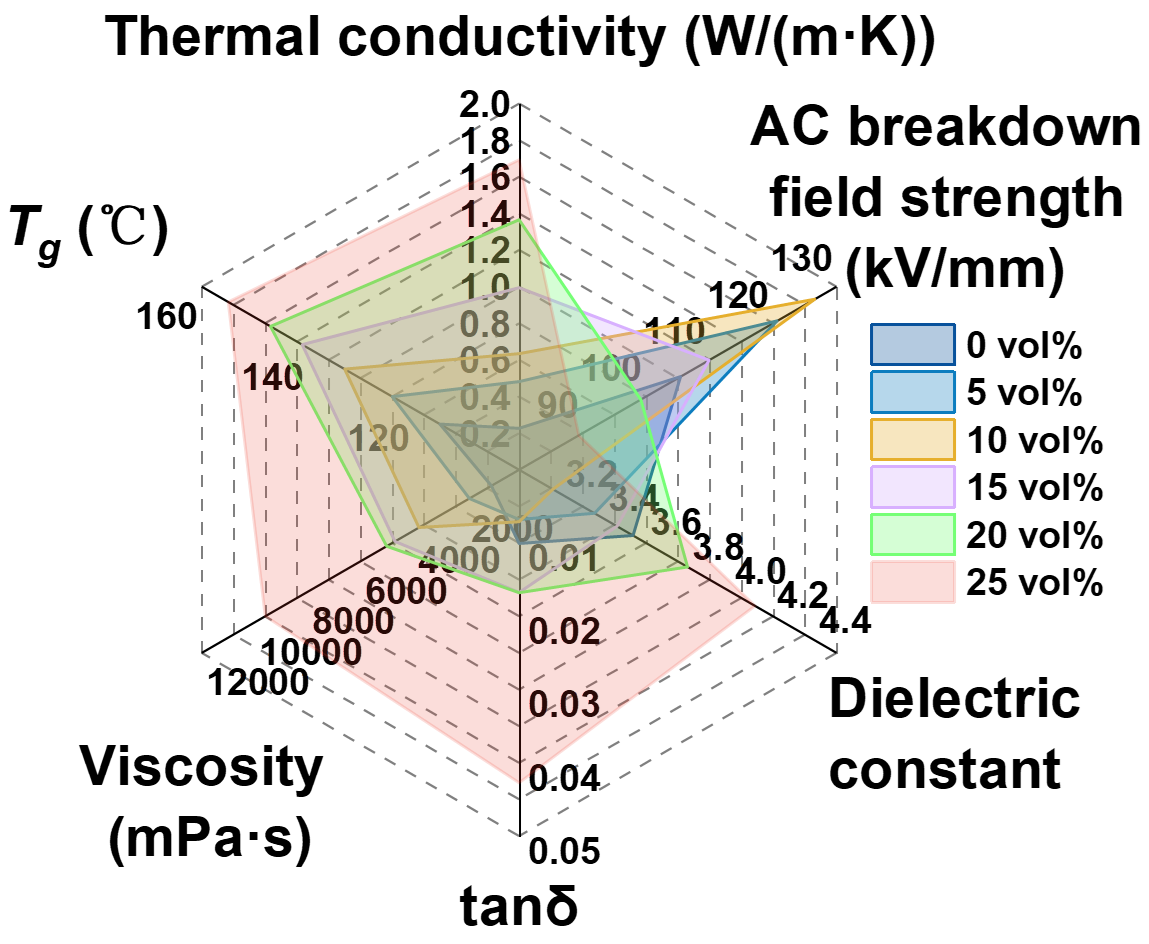


**Figure S5** Radar chart of BN@G-POSS/EP under various concentrations.

**Figure S6** Diagram of the devices for partial discharge test.


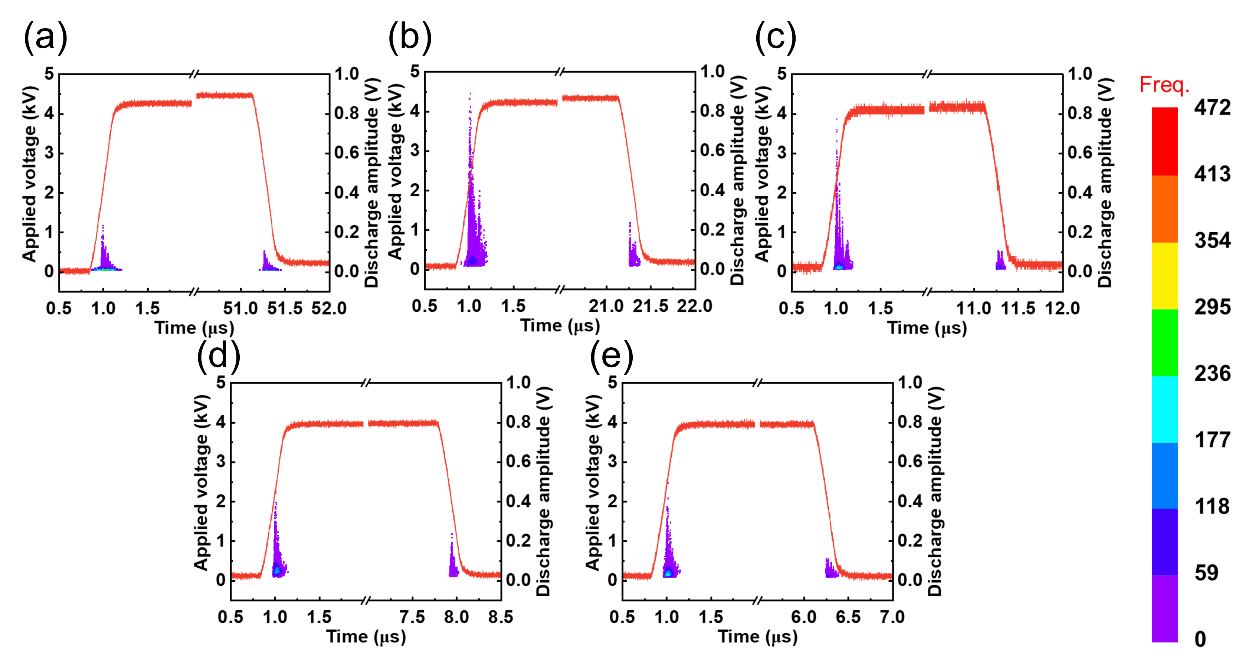


**Figure S7** Discharge amplitude vs time under the frequency of (a) 10kHz, (b) 25kHz, (c) 50kHz, (d) 75kHz, (e) 100kHz for pristine EP.

**Figure S8** Discharge amplitude vs time under the frequency of(a) 10kHz, (b) 25kHz, (c) 50kHz, (d) 75kHz, (e) 100kHz for BN@G-POSS/EP.


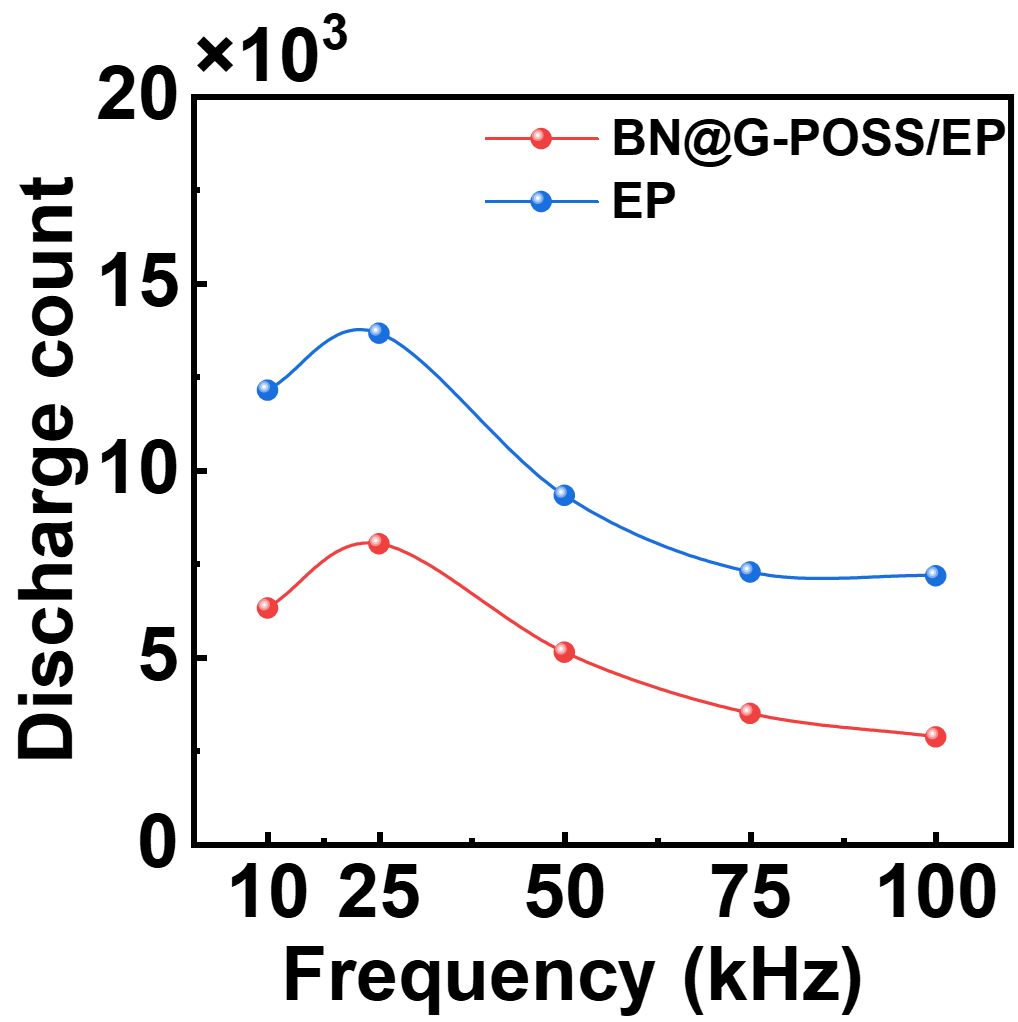


**Figure S9** Discharge count vs time under the frequency of 10kHz, 25kHz, 50kHz, 75kHz, 100kHz for EP and BN@G-POSS/EP.

**Figure S10** Discharge amplitude vs time under the rise time of (a) 100ns, (b) 200ns, (c) 300ns, (d) 400ns, (e) 500ns for pristine EP.

**Figure 11** Discharge amplitude vs time under the rise time of (a) 100ns, (b) 200ns, (c) 300ns, (d) 400ns, (e) 500ns for BN@G-POSS/EP.


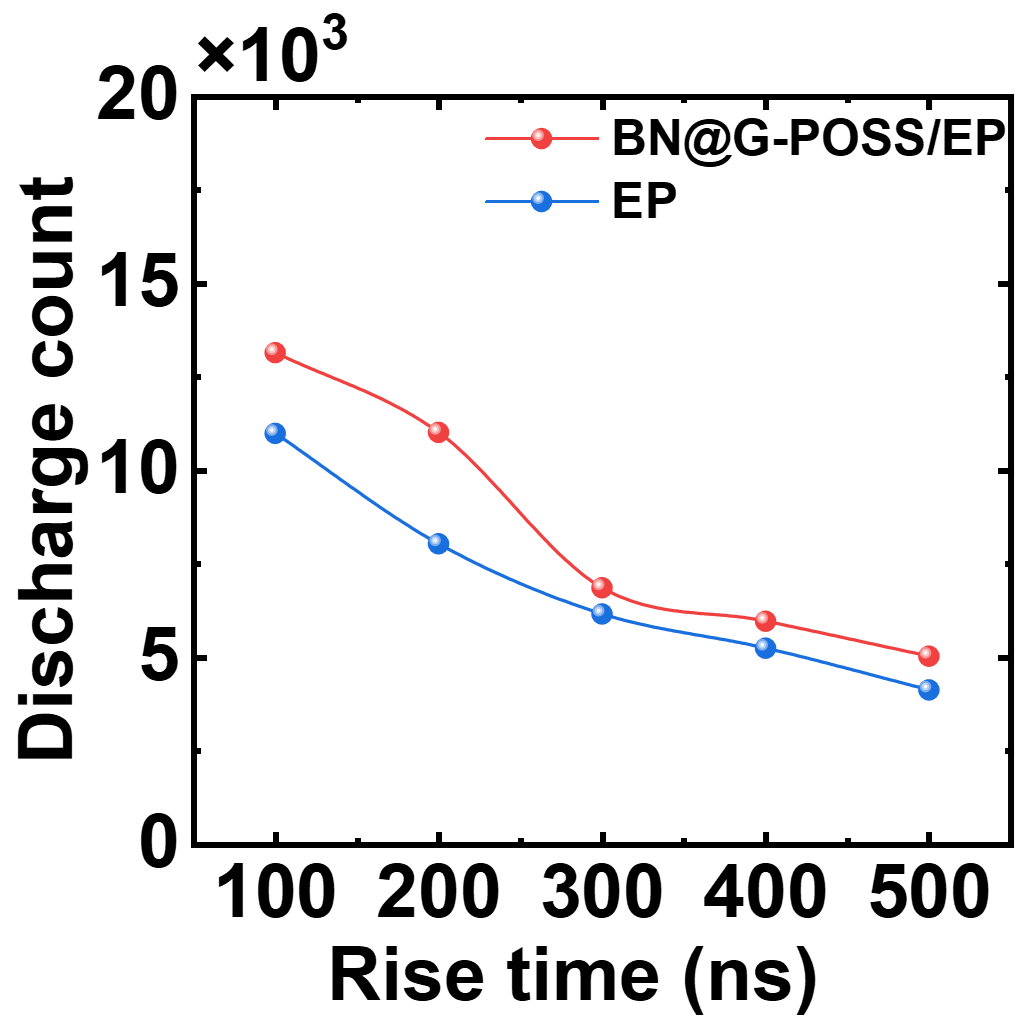


**Figure S12** Discharge count vs time under the rise time of 100ns, 200ns, 300ns, 400ns, 500ns for EP and BN@G-POSS/EP.

**Figure S13** Discharge amplitude vs time under the temperature of (a) 20℃, (b) 80℃, (c) 110℃, (d) 140℃, for pristine EP.

**Figure S14** Discharge amplitude vs time under the temperature of (a) 20℃, (b) 80℃, (c) 110℃, (d) 140℃, for BN@G-POSS/EP.


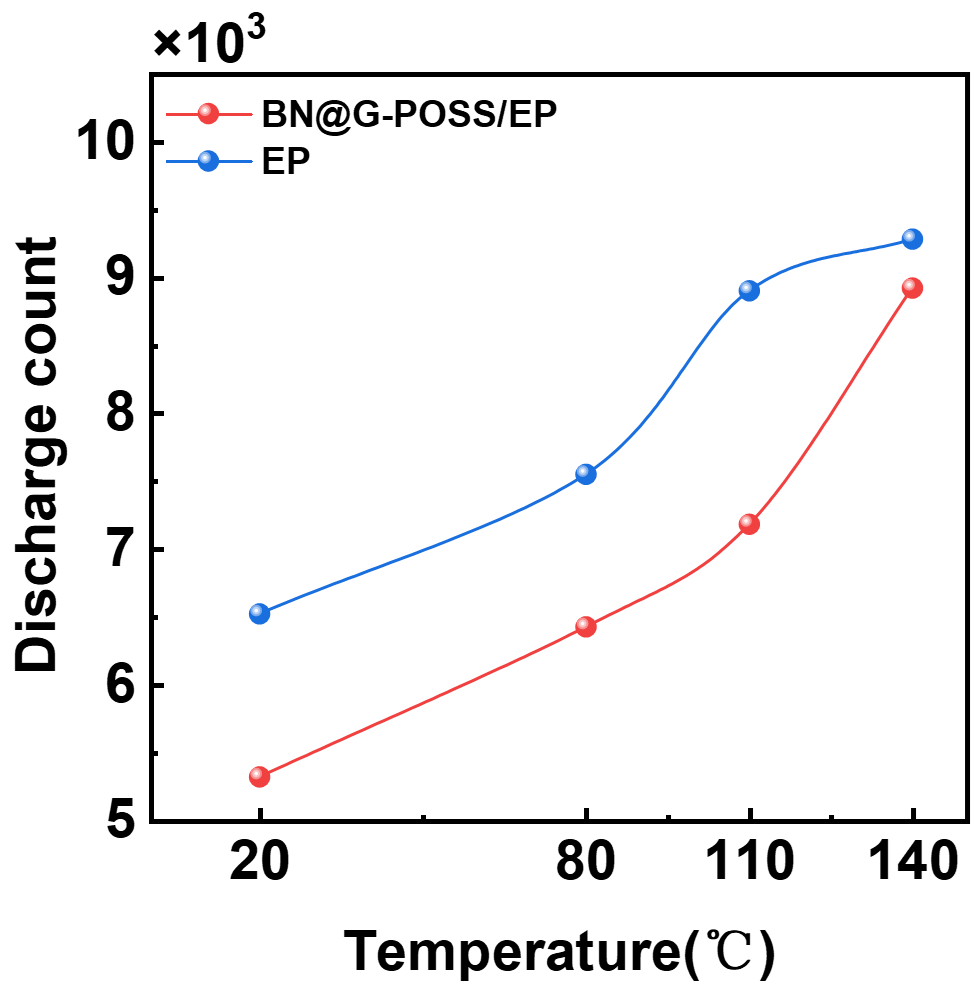


**Figure S15** Discharge count vs time under the temperature of 20℃, 80℃,110℃, 140℃, for EP and BN@G-POSS/EP.


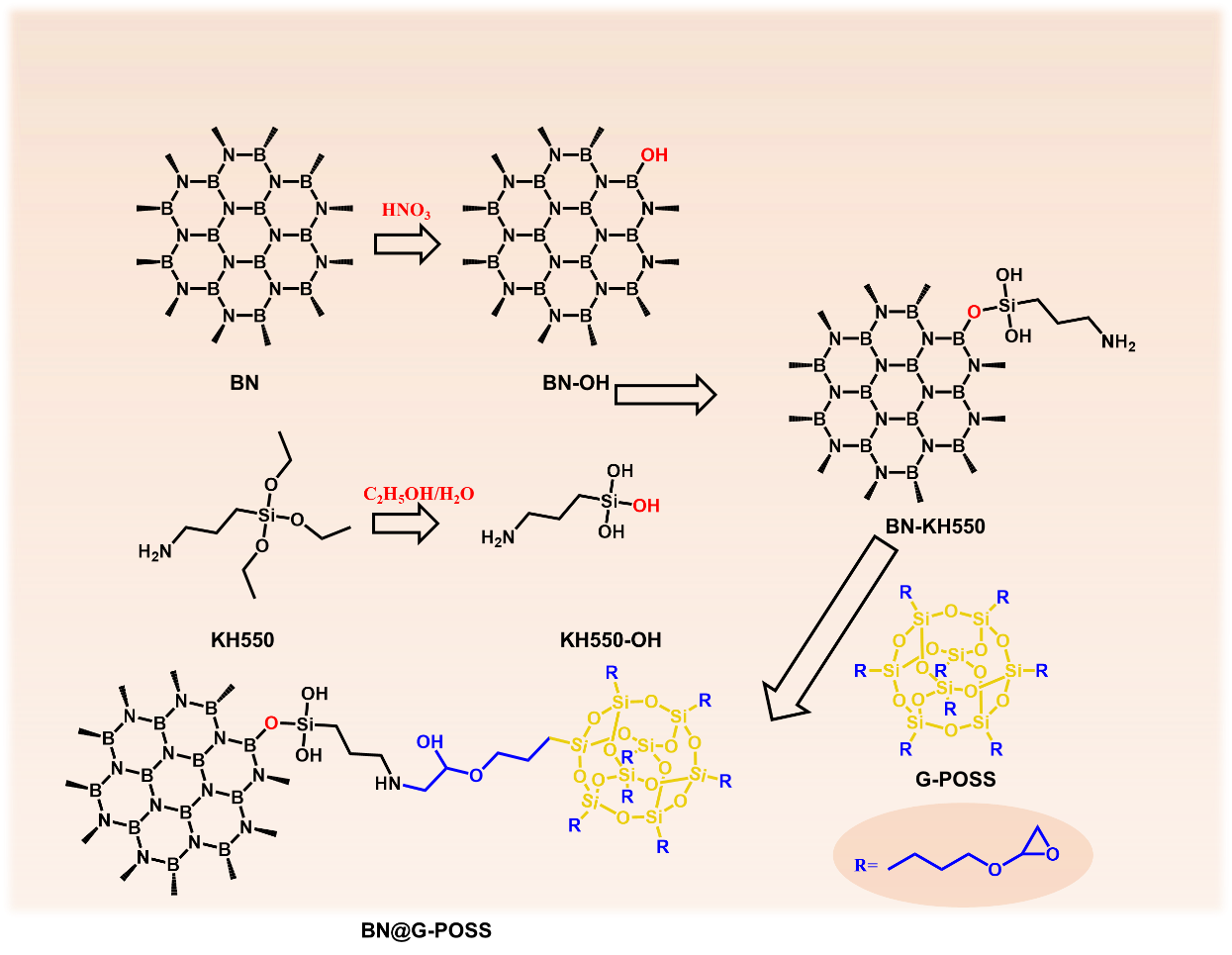


**Figure S16** Surface functionalization mechanism of BN@G-POSS.

**Figure S17** Digital photos of EP, BN/EP and BN@G-POSS/EP composite.

**Figure S18** Process flow chart for the preparation of BN@G-POSS/EP.

Table S1 Breakdown strength and dielectric loss comparison

| Material | Breakdown strength(kV/mm) | Dielectric loss (≥100kHz 30 ℃) | Ref. |
| --- | --- | --- | --- |
| BN/EP(5wt%) | 35.2 | 0.015(100kHz) | [R1] |
| EP/POSS(5wt%) | 33.71 | 0.04(1MHz) | [R2] |
| EP/gIycidyi POSS(4.8wt%)  BN@G-POSS/EP | 62  59.09 | -  0.014(100kHz) | [R3]  This work |

‘Benchmarked against other representative materials, the proposed composite exhibits a significantly enhanced breakdown strength compared to R1 (BN/EP) and R2 (EP/POSS). Furthermore, while R3 (EP/glycidyi POSS) suffers from a pronounced upward drift in low-frequency dielectric loss and lacks corresponding high-frequency data, this work successfully achieves a synergistic optimization. It dramatically boosts the breakdown strength while fully preserving excellent dielectric loss stability at high frequencies, thereby demonstrating a distinct performance advantage.’

Table S2 Summary table of the PD performance metrics

| Frequency  (kHz) | d*v*/d*t*  (ns) | Temperature  (℃) | PDIV  (V) | Discharge count  (times) | Total discharge amplitude(V) |
| --- | --- | --- | --- | --- | --- |
| 10 | 200 | 25 | 3285.7 | 6318 | 120.86499 |
| 25 | 200 | 25 | 3342.8 | 8036 | 228.78004 |
| 50 | 200 | 25 | 3422.86 | 5136 | 154.52515 |
| 75 | 200 | 25 | 3493.6 | 3498 | 148.95702 |
| 100 | 200 | 25 | 3523.6 | 2866 | 128.78403 |
| 25 | 100 | 25 | 3185.7 | 10990 | 397.9636 |
| 25 | 200 | 25 | 3342.8 | 8036 | 228.78004 |
| 25 | 300 | 25 | 3462.86 | 6160 | 147.51953 |
| 25 | 400 | 25 | 3533.6 | 5242 | 115.91406 |
| 25 | 500 | 25 | 3623.6 | 4123 | 94.17086 |
| 10 | 100 | 20 | 3201.4 | 5320 | 325.4 |
| 10 | 100 | 80 | 3077.3 | 6426 | 618.71994 |
| 10 | 100 | 110 | 2973.8 | 7180 | 1070.74522 |
| 10 | 100 | 140 | 2625.2 | 8920 | 2010.77 |

**References**

[R1] Y. Tang, P. Zhang, M. Zhu, J. Li, Y. Li, Z. Wang, Huang, L, *Materials* **2019**, 12, 4112.

[R2] X. Huang, Y. Li, F. Liu, P. Jiang, T. Lizuka, K. Tatsumi, *IEEE Transactions on Dielectrics and Electrical Insulation*, **2014**, 21, 004314.

[R3] M. Takala, M. Karttuen, J. Pelto, P. Salovaara, T. Munter, M. Honkanen, *IEEE Transactions on Dielectrics and Electrical Insulation*, **2008**, 15, 4656229.
